# Correction: The neuroelectric dynamics of the emotional anticipation of other people’s pain

**DOI:** 10.1371/journal.pone.0211667

**Published:** 2019-01-29

**Authors:** The PLOS ONE Staff

## Notice of Republication

This article was republished on January 18, 2019 to correct errors with the presentation of the *eta* symbol that were introduced during the typesetting process. The publisher apologizes for the errors. Please download this article again to view the correct version. The originally published, uncorrected article and the republished, corrected articles are provided here for reference.

## Supporting information

S1 File Originally published, uncorrected article. (PDF)

S2 File Republished, corrected article. (PDF)
