# Supplementary material for: The neuroelectric dynamics of the emotional anticipation of other people’s pain
Source: PLoS One. 2018 Aug 1;13(8):e0200535. doi: 10.1371/journal.pone.0200535 (PMC6070195; doi:10.1371/journal.pone.0200535)
Supplement: S1 File — (Figure A) Example of behavioral results for one participant in response to the VideoEI90% in the preliminary experiment. This participant presented a significant memory displacement (PSE = 64.8%, further from 90%). In abscissa, the gray brackets illustrate the three TestEIs triplets shown to this participant in the main experiment. In this example, the “Expected Incongruent TestEIs” triplets were equal to 25%, 30%, 35% and 95%, 100%, 105%. The “expected Congruent TestEIs” triplet was equal to 60%, 65% and 70%. (DOCX) [file pone.0200535.s001.docx]

**Preliminary experiment (Experiment 1)**

The aim of the preliminary experiment was to determine which TestEI stimuli should be presented to participants in the subsequent main experiment: they would be recognized as equal to or different from their memorized intensity.

**Materials and methods**

**Participants**

25 participants (15 women, 10 men; *M* = 24.5 years-old, SD = 4.8), with normal or corrected-to-normal vision, took part in this experiment. All participants gave their informed written consent before the experiment, in accordance with the ethical standards of the Declaration of Helsinki. The EA 4532 local Ethics Committee of Université Paris-Sud approved this study for both experiments.

**Stimuli**

The videos were the same as those described for the main experiment: VideoEI50% and VideoEI90%. There were nine TestEIs, ranging from -40% to +40% around the VideoEI value, in 10% stages. Namely, the TestEIs ranged from 10% to 90% for VideoEI50% and from 50% to 130% (in 10% stages), for VideoEI90% (see Fig 2 for a trial with VideoEI90% and TestEI65%).

**Procedure**

The trial sequence and the VideoEI conditions (50% and 90%) were the same as in the main experiment (see Fig 2). However, the instructions and the TestEIs differed in the preliminary experiment. Depending on the trials, the final expression intensity was either 50% (VideoEI50%), or 90% (VideoEI90%). Participants had to indicate whether the TestEI was either “less” or “more” intense than the final VideoEI. They responded by pushing the corresponding keyboard key (less = escape; more = Enter)

**Behavioral data analysis**

A logistic cumulative distribution function was matched to the proportions of the “more intense” answers as a function of the TestEI stimuli in order to estimate the Point of Subjective Equality (PSE) (see Fig 1 for an illustration). PSE1 (PSE in this preliminay experiment) provided an estimate of the TestEI stimuli, for which participants would answer “more intense” and “less intense” with equal .50 probability. It reflected participants’ memorized final expression intensity (Jarraya et al. 2005). The PSE1 values were computed for each participant and each VideoEI condition (50% and 90%). The memory bias corresponded to the difference between PSE1 and the actual final VideoEI value (50% or 90%). Student’s *t* tests were then used to test the memory bias for each VideoEI condition, as well as between conditions.

**Results**

Results indicated a non significant memory bias (*M* = 3.8%, SD = 12.0%, ranging from -21.3% to +28.8%) for the VideoEI50% condition, *t*(24) = 1.6, *p* = .132342. In contrast, there was a significant, negative memory bias (*M* = -21.1%, SD = 12.6%, ranging from -55.1% to -3.5%) for the VideoEI90% condition, *t*(24) = -8.20087, *p* = 0,00000002. Memory displacement (bias values) varied significantly with VideoEI conditions (50% and 90%), *t*(24) = 9.8, *p* = .0000000007.

**Selection of stimuli for the main experiment**

Ahead of the main experiment, for each participant, three TestEI triplets of stimuli (see S1 Fig. A, abscissa) were defined on the basis of their individual PSE (see S1 Fig. A). These were computed in the preliminary experiment for both the VideoEI50% and VideoEI90% conditions. One TestEI triplet was expected to be perceived by participants as being similar to her/his memorized intensity in the main experiment, i.e., assuming that the PSE in both experiments are equal. This “Expected Congruent TestEI” triplet was centered on each participant’s PSE, and included the PSE, PSE +5% and PES -5% TestEIs (S1 Fig. A). Two other TestEI triplets were expected to be perceived as being different with respect to their memorized intensity in the main experiment. These “Expected Incongruent TestEIs” triplets were either smaller or greater than PSE by an amount of 30%, 35% and 40% (S1 Fig. A). As a consequence, TestEIs in the main experiment varied with participants.


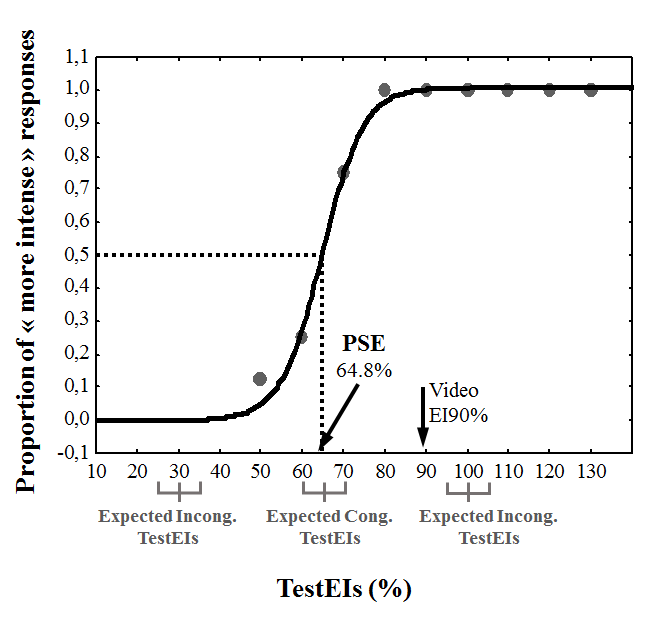


**Figure A (Experiment 1). Example of behavioral results for one participant in response to the VideoEI90% in the preliminary experiment.** This participant presented a significant memory displacement (PSE = 64.8%, further from 90%). In abscissa, the grey brackets illustrates the three TestEIs triplets shown to this participant in the main experiment. In this example, the “Expected Incongruent TestEIs” triplets were equal to 25%, 30%, 35% and 95%, 100%, 105%. The “expected Congruent TestEIs” triplet was equal to 60%, 65% and 70%.
