# Supplementary material for: The neuroelectric dynamics of the emotional anticipation of other people’s pain
Source: PLoS One. 2018 Aug 1;13(8):e0200535. doi: 10.1371/journal.pone.0200535 (PMC6070195; doi:10.1371/journal.pone.0200535)
Supplement: S2 File — (Figure A) Initial path analysis mediation model testing for the spatio-temporal unfolding of the mismatch effect from Occipital P100 up to Left Occipital LPP. A: this initial model tested the mismatch effect at three components as predictors of the mismatch effect at Left_O_LPP using a serial multiple mediator model with two mediators (F_N170 and T_N170). B: the second model introduced the mismatch effect at F_LPP while considering two simple mediation models arranged in series, with T_N170 and Left_O_LPP as dependent variables. Standardized (β) coefficients (in bold) are provided together with unstandardized coefficients (between parentheses). The unstandardized coefficients are in μV unit, together with significance level of effects as §p < .10, *p < .05, ** p < .01, *** p < .001. Significant indirect effects are illustrated with thicker lines. (DOCX) [file pone.0200535.s002.docx]

**Path analysis model testing**

The initial model (illustrated in S2 Fig. A, A) was a serial multiple mediator model (see [51], p.143 and after) with two mediators, namely the mismatch effect at Frontal N170 (F_N170) and Temporal N170 (T_N170). The results showed that in spite of a significant, (*p* = .042) Total effect (Direct effect + Indirect effects) of O_P100 on Left_O_LPP, partitions into direct and indirect components only evidenced a marginal (*p* = .084) direct contribution of O_P100 on Left_O_LPP. None of the indirect effects taken individually (O_P100 ➔ F_N170 ➔ left_O_LPP with BootCI = [-.187; .405]; O_P100 ➔ F_N170 ➔ T_N170 ➔ left_O_LPP with BootCI = [-.373; .089]; O_P100 ➔ T_N170 ➔ left_O_LPP with BootCI = [-.548; .153]) were significant, nor did the Total indirect effect (estimated as the sum of all the specific indirect effects) reach significance (BootCI = [-.579; .267]). However, given the significant local effects between O_P100, F_N170, and T_N170 (see S2 Fig. A, A), a follow-up analysis was conducted testing the hypothesis of a feedforward effect of the O ROI on both the F ROI and the T ROI, together with a feedback effect from the F ROI on the T ROI, along the lines of [79]. The results showed a significant Total effect of the O ROI on the T ROI, *p* = .0008, with significant Direct (O ROI ➔ T ROI), *p* = .012, and Indirect (O ROI ➔ F ROI ➔ ROI) components (BootCI = [.031; .705]). Building on this last result, a second model was tested in which T_N170 was considered as the input (see S2 Fig. A, B).


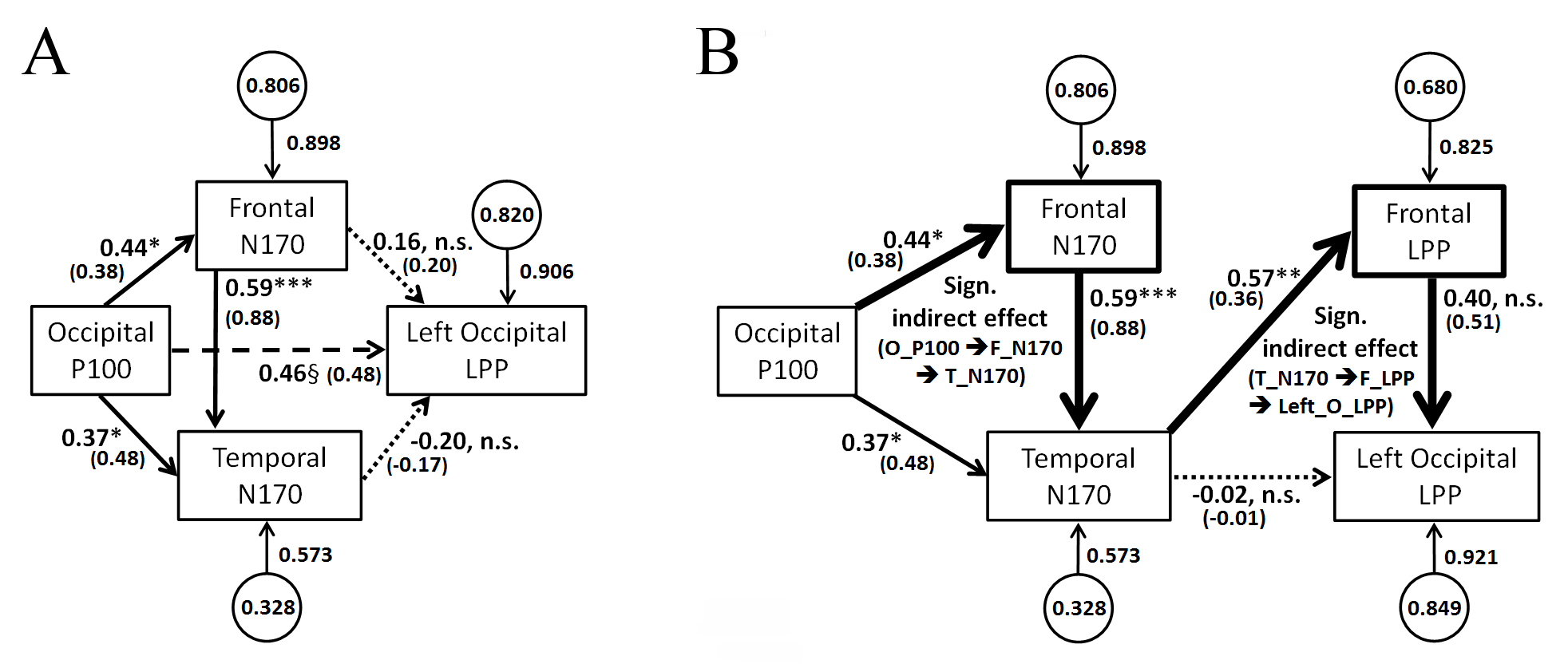


**Figure A. Path analysis model testing. () Initial path analysis mediation model testing for the spatio-temporal unfolding of the mismatch effect from Occipital P100 up to Left Occipital LPP.** A: this initial model tested the mismatch effect at three components as predictors of the mismatch effect at Left_O_LPP, using a serial multiple mediator model with two mediators (F_N170 and T_N170). B: the second model introduced the mismatch effect at F_LPP while considering two simple mediation models arranged in series, with T_N170 and Left_O_LPP as dependent variables. Standardized (β) coefficients (in bold) are provided together with unstandardized coefficients (between parentheses). The unstandardized coefficients are in µV unit, together with significance level of effects as §*p* < .10, **p* < .05, ** *p* < .01, *** *p* < .001. Significant indirect effects are illustrated with thicker lines.

The second model involved two simple mediation models arranged in series. The first simple mediation model (see previous paragraph for results) considered F_N170 as a mediator and T_N170 as the intermediate dependent variable, whereas the second simple mediation model considered used T_N170 as an input and Left_O_LPP as the dependent variable, with the mismatch effect at the Frontal LPP (F_LPP) as a mediator. The results showed that the mismatch effect at Left_O_LPP was accounted for mainly by the significant “T_N170 ➔ F_LPP ➔ Left_O_LPP” indirect effect (BootCI = [.009; .503]), as illustrated in S2 Fig. A, B. This mediation by the Frontal LPP is supposedly consistent with the LORETA findings, showing the involvement of the Medial Frontal Gyrus (BA10) in the mismatch effect at the LPP time window.
