# Supplementary material for: Correction: The neuroelectric dynamics of the emotional anticipation of other people’s pain
Source: PLoS One. 2019 Jan 29;14(1):e0211667. doi: 10.1371/journal.pone.0211667 (PMC6350995; doi:10.1371/journal.pone.0211667)
Supplement: S2 File — (PDF) [file pone.0211667.s002.pdf]

PLOS ONE

RESEARCH ARTICLE

The neuroelectric dynamics of the emotional anticipation of other people's pain

Dorian Dozolme[1,2]*, **Elise Prigent**[3], **Yu-Fang Yang**[1,2], **Michel-Ange Amorim**[1,2]

1 CIAMS, Univ. Paris Sud, Université Paris-Saclay, France, **2** CIAMS, Université d'Orléans, Orléans, France, **3** LIMSI, CNRS, Univ. Paris-Sud, Université Paris-Saclay, Orsay, France

* dozolmedorian@gmail.com

Check for updates

OPEN ACCESS

Citation: Dozolme D, Prigent E, Yang Y-F, Amorim M-A (2018) The neuroelectric dynamics of the emotional anticipation of other people's pain. PLoS ONE 13(8): e0200535. https://doi.org/10.1371/journal.pone.0200535

Editor: Cosimo Urgesi, Universita degli Studi di Udine, ITALY

Received: October 4, 2017

Accepted: June 28, 2018

Published: August 1, 2018

Copyright: © 2018 Dozolme et al. This is an open access article distributed under the terms of the Creative Commons Attribution License, which permits unrestricted use, distribution, and reproduction in any medium, provided the original author and source are credited.

Data Availability Statement: All relevant data are within the paper and its Supporting Information files.

Funding: The reported research was funded by a grant from Agence Nationale de la Recherche, EMCO 2011 (COMPARSE project) to M-AA. The funders had no role in study design, data collection and analysis, decision to publish, or preparation of the manuscript.

Competing interests: The authors have declared that no competing interests exist.

Abstract

When we observe a dynamic emotional facial expression, we usually automatically anticipate how that expression will develop. Our objective was to study a neurocognitive biomarker of this anticipatory process for facial pain expressions, operationalized as a mismatch effect. For this purpose, we studied the behavioral and neuroelectric (Event-Related Potential, ERP) correlates, of a match or mismatch, between the intensity of an expression of pain anticipated by the participant, and the intensity of a static test expression of pain displayed with the use of a representational momentum paradigm. Here, the paradigm consisted in displaying a dynamic facial pain expression which suddenly disappeared, and participants had to memorize the final intensity of the dynamic expression. We compared ERPs in response to congruent (intensity the same as the one memorized) and incongruent (intensity different from the one memorized) static expression intensities displayed after the dynamic expression. This paradigm allowed us to determine the amplitude and direction of this intensity anticipation by measuring the observer's memory bias. Results behaviorally showed that the anticipation was backward (negative memory bias) for high intensity expressions of pain (participants expected a return to a neutral state) and more forward (memory bias less negative, or even positive) for less intense expressions (participants expected increased intensity). Detecting mismatch (incongruent intensity) led to faster responses than detecting match (congruent intensity). The neuroelectric correlates of this mismatch effect in response to the testing of expression intensity ranged from P100 to LPP (Late Positive Potential). Path analysis and source localization suggested that the medial frontal gyrus was instrumental in mediating the mismatch effect through top-down influence on both the occipital and temporal regions. Moreover, having the facility to detect incongruent expressions, by anticipating emotional state, could be useful for prosocial behavior and the detection of trustworthiness.

Introduction

Humans possess the capacity of automatically and unconsciously anticipating the following movement of an observed movement that could be an object [1], a scene [2], a body or a facial

expression [3–5]. Indeed, individuals can perceive the facial expressions of other people in order to gain an immediate impression of another individual's current and future emotional state of mind [6]. The processes used are highly adaptive to meet the demands of a world in which we are constantly required to react to, and often anticipate, the behavior of others [7]. We have chosen to focus on the perception of pain behavior in other people since the ability to recognize and interpret other people's pain quickly can be of great importance to both the person who is suffering and the observer [8]. In addition, it is critical to everyday social functioning [9].

Pain is both a specific sensation (i.e., a reflexive reaction of the body) and an emotional state [10]. Individuals who experience pain often adopt pain behaviors [11], either to attenuate their own pain (e.g., by touching or guarding) or to communicate pain to others (through words, sounds and facial expressions). Studies of the production of facial pain expressions have shown that they appear to be specifically adapted to social communication [12] and have a survival function by demanding attention and prioritizing escape, recovery and healing [13]. In fact, "suffering offers us the best protection for survival" [14]. Moreover, perceiving the pain of others is also supported by a complex response in the observer's corticospinal system that may allow a freezing or escaping reaction [15]. Studies of pain perception have highlighted that, in order to estimate other people's pain levels, individuals assign greater importance to facial expressions than to body language [16, 17]. In the same way, facial expressions are assigned a greater importance than verbal information in order to discriminate between genuine, suppressed and faked pain [18]. That is why, among different pain behavior, we focused our study on the perception of facial pain expression behavior and we investigated the emotional anticipation of dynamic facial pain expressions from both a behavioral and neurocognitive approach.

The perception of facial expressions of emotion has mostly been studied with the use of static stimuli such as photographs; in real life, however, people's facial expressions of emotion are dynamic. Some studies have shown that facial expression recognition can be improved by employing dynamic (rather than static) information [19, 20]. The recognition of subtle (i.e., barely intense) dynamic facial expressions of emotion has proved more effective compared with the identification of subtle static facial expressions [21]. This difference in perception between static and dynamic facial pain expressions may be explained by the human capacity to anticipate and extrapolate a dynamic movement, such as a dynamic facial expression [22].

People automatically anticipate the future of a movement, which implies memorization of the final position of a moving target often displaced forward in the direction of target motion. Such memory displacement or memory bias has been termed "representational momentum"; namely, a second-order isomorphism between physical and representational inertia [3]. More specifically, the memorized intensity of an emotional facial expression may obey such a memory bias effect. Yoshikawa and Sato [4] reported a forward representational momentum effect for dynamic facial expressions of fear, joy, disgust, sadness, anger and surprise (ranging from neutral to maximal emotional intensity), with a memorized intensity superior to the final intensity displayed by the participant (associated to a positive memory bias). However, memory displacements may also be backward and oriented toward the emotionally neutral state (associated to a negative memory bias) [23, 24]. Some authors have shown additional evidence relating to representational momentum when perceiving facial expressions of emotion [6, 25]. When investigating dynamic facial expressions from emotional to neutral, they found that final neutral expressions were judged as presenting an emotional valence opposite to the initial one. For example, Jellema et al. [25] reported that when an expression, that moved backward from joy to neutral, was presented and disappeared, the final neutral expression remembered was a slightly angry one. On the other hand, an expression that moved backward from anger

to neutral was remembered as being slightly happy. These findings were interpreted in terms of "emotional anticipation", which can be defined as the ability to involuntarily anticipate how an agent's emotional state of mind will develop in the immediate future, based on the immediate perceptual history. This emotional anticipation may reflect top-down emotional processes [25] and constitute a low-level form of the mind-reading mechanism [6]. According to embodiment and simulation theories, the perception of other people's facial expressions [26, 27], specifically those of pain [28], is associated with automatic and unconscious processes such as the involuntary motor simulation of the observed action. Accordingly, other lines of research have investigated neural correlates of emotional anticipation of the pain of others, by using different physiological techniques, such as single pulse TMS (transcranial magnetic stimulation) to induce MEPs (motor-evoked potentials). Some of these studies [15, 29–31] evidenced inhibition of specific MEPs while viewing a needle hurting a human body part, compared to non-painful conditions. Such an inhibition while viewing a painful stimulus on others supports the "mirror-matching" simulation theory. It is worth noting that these authors did not study pain expressions but painful stimuli on a body part. However, another study [32] reported a similar MEP inhibition while using fearful, happy and neutral body expressions as stimuli. Finally, the sensorimotor structures involved in empathy for pain were also investigated using somatosensory-evoked potentials (SEPs). For example, Bufalary et al. [33] administered non-painful electrical stimulations to one of the participants' hands while stimuli similar to the one used by Avenanti et al. [15, 29, 30] and Bucchioni et al. [31] were displayed. SEPs recorded from the primary somatosensory cortex (S1) showed an increased P45 component (reflecting activity in S1) while participants viewed the painful stimuli, whereas the SEP decreased while they viewed (touched) non-painful stimuli. These results suggest that S1 might contribute to the "mirror-matching" simulation of the pain of others. Neurocognitive literature has emphasized the distributed nature of the areas of the brain which contribute to facial perception. Changeable features, such as facial expressions, relate to visual codes in the Superior Temporal Sulcus (STS). They also involve the fronto-parietal activation of emotion representation and motor programs in the mirror neuron system responsible for producing expressions [34]. In relation to expressions of pain, Reicherts et al. [35] used ERPs to support the idea of the prioritized processing (with enhanced Late Positive Potentials, LPPs) of painful dynamic facial expressions when compared with joyful (and possibly fearful) dynamic expressions, as well as neutral dynamic expressions. The perception of dynamic facial expressions triggers automatic low-level processes involved in the prediction of forthcoming expressions [25]. There is evidence that dynamic facial expressions are represented as anticipated motion trajectories in the visual (fusiform gyrus, STS) and premotor areas [36], with their post-stimulus onset as early as 165 ms to 237 ms. These findings, as well as those regarding mirror-matching in sensorimotor brain areas, are consistent with the 'extended' mirror neuron system proposed by Pineda [37] containing shared representations for action and emotion, automatically retrievable during the observation of others.

In our study, we conducted behavioral measures and ERPs to investigate the neurocognitive mechanisms involved in the emotional anticipation of facial expressions of pain. For this, we used a representational momentum paradigm inspired by the work of Courgeon et al. [23] and Thornton [24]. In this paradigm, a static expression was displayed after a dynamic expression (video), separated by a mask. Participants had to memorize the final intensity of the dynamic expression (the last frame of the video) and compare it to a static test expression intensity (TestEI). As previously mentioned, by using this paradigm, if a memory bias of the final expression intensity is measured, it supposes the participant's automatic anticipation of the immediate future of the expression. In a preliminary experiment (see S1 File), we measured this memory bias (amplitude of the anticipation) for each participant. This allowed us, in the present study,

to adapt static test expression intensity (TestEI) as a function of the participants' memory bias, by displaying either TestEI equal (congruent trials) or different (incongruent trials) from the intensity memorized, and therefore anticipated, by the participant. So, independently of the anticipation amplitude of the future of the expression, we displayed a static expression intensity (TestEI) to the participant, congruent or incongruent to the intensity they were expecting. In other words, a static expression different from the expected intensity can be considered as an incongruent intensity, and a static expression equal to the expected intensity can be considered as a congruent intensity by the participant. Our objective was to study a neurocognitive biomarker of anticipatory processes for facial pain expressions, operationalized as a mismatch effect (i.e., difference in ERP between congruent and incongruent trials). Our hypothesis here was that, even if participants were instructed to memorize the state of a dynamic expression at the end of the video, rather than anticipate how the expression would develop, pain anticipation mechanisms were expected to modulate both behavioral (Reaction Time, RT) and physiological (ERP) correlates of mismatch detection when the memorized state of the dynamic expression was to be compared with a static image of pain expression.

Materials and methods

Participants

The ERP data of 25 individuals (15 women, 10 men; *M* = 24.5 years-old, SD = 4.8) participating in a preliminary behavioral study (see S1 File for details), were analyzed.

Participants had normal or corrected-to-normal vision. All participants gave informed written consent before the experiment, in accordance with the ethical standards of the Declaration of Helsinki. The EA 4532 local Ethics Committee of Paris-Sud University approved this study for both experiments.

Stimuli

A facial expression is characterized by the type and number of facial muscles contracted and the intensity with which they contract [38]. The Facial Action Coding System, known as FACS [38], provides a framework for the description of facial expressions in terms of Action Units of the face (AUs). An observable AU results from the contraction of one or a group of muscle(s) and the different facial expressions that result from the activation of one or several AU(s). The facial expression of pain recruits three main AUs: AU 4, which corresponds with brow lowering; AUs 6 and 7, which correspond with orbit tightening; and AUs 9 and 10, which correspond with levator contraction [39, 40]. In the present study, we used a facial expression of pain that requires the activation of the following facial AUs: AU 4, AUs 6 and 7, and AUs 9 and 10.

Dynamic stimuli were created with the help of realistic 3D synthetic facial movements, aimed at mobilizing specific Action Units (AUs) of pain, using 3ds Max 2010® software. A virtual character was imported from the Poser 8® software library. For this virtual character, a dynamic facial expression of pain was created, which recruited five principal AUs of pain, based on the FACS [38]. In accordance with Oliveira et al. [40], these AUs were: *brow lowering* (AU 4), *cheek raise* (AU 6), *lid tightening* (AU 7), *nose wrinkling* (AU 9) and *upper raise* (AU 10). We created a facial pain expression composed of all five AUs with an intensity of 100% (see Fig 1), which was arbitrarily defined and considered as a naturally high facial pain expression (according to Ekman & Friesen [38] model). Using 3ds Max 2010® software, we carried out morphing in equal stages to create the intermediary intensities between neutral expression (by default in the software) and 100% intensity. Two videos of two different intensities were generated: 50% and 90% of Emotional Intensity (EI). These two videos corresponded to the

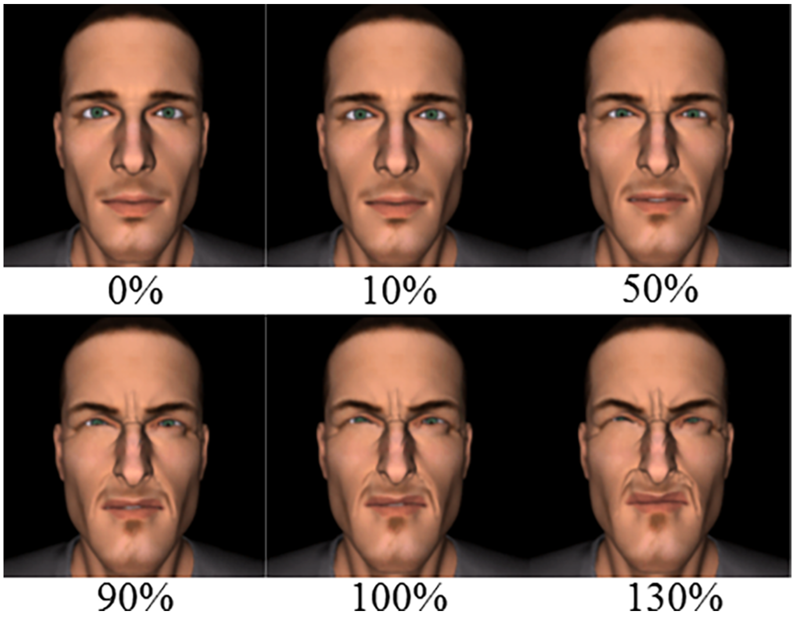

Fig 1. Illustration of expression intensity displayed during the experiment, ranging from 0% (neutral) to 130%. Morphing at equal stages was used to create intermediary intensities between a neutral (0%) and a naturally high facial expression of pain (100%). Higher intensities were also created (up to 130%), which gave more caricatured expressions.

https://doi.org/10.1371/journal.pone.0200535.g001

VideoEI50% and VideoEI90% conditions used in both the preliminary and main experiments. Static expressions were also created at different pain intensities, from 10% to 130%, which corresponded to the Test Expression Intensity (TestEI). We set 100% as the maximal natural expression intensity target; thus, TestEIs greater than 100% gave more caricatured expressions.

TestEIs were defined on an individual basis in a preliminary experiment (See S1 File). For each participant, three TestEI triplets of stimuli (see Figure A in S1 File, abscissa) were defined on the basis of their individual memory bias, corresponding to their Point of Subjective Equality, PSE (see Figure A in S1 File), computed in the preliminary experiment, in each video

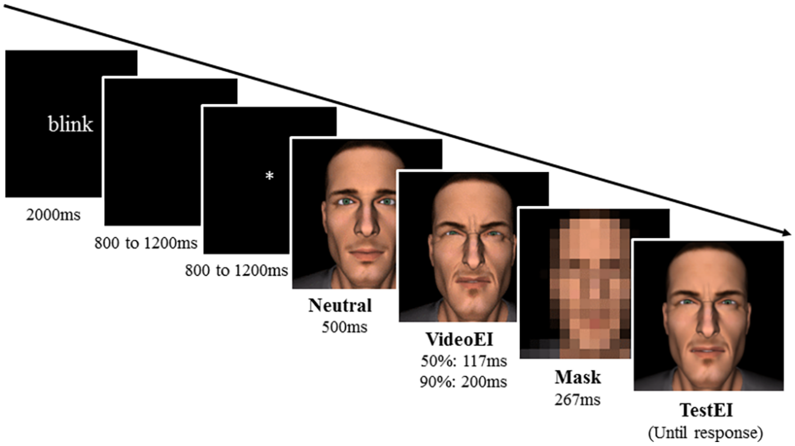

Fig 2. Illustration of a trial sequence. Here, the video reached 90% intensity (VideoEI90%) and the TestEI presented 65% pain intensity. The participants had to memorize the final expression intensity (90%) and compare this memorized expression with the TestEI. The static TestEI was displayed until the participants had given their responses via the keyboard.

https://doi.org/10.1371/journal.pone.0200535.g002

condition. Assuming that the PSE in both experiments would be equal, we expected one "Expected Congruent TestEI" triplet to be perceived as being of a similar intensity to that of the final video: it was centered on the participants' PSE, and included the PSE, PSE +5% and PES -5% TestEIs of the preliminary experiment (Figure A in S1 File). In contrast, the "Expected Incongruent TestEIs" triplets (either smaller or greater than PSE by an amount of 30%, 35% and 40%) were expected to be perceived as different with respect to their memorized intensity in the main experiment.

All stimuli (i.e., videos and static images) were made up of 150 x 150 pixels and displayed on a 1024 x 768 pixels 60 Hz screen at a 57 cm viewing distance. They subtended a 4° x 4° visual angle. TestEIs could differ between participants because they were selected as a function of the individuals' memory bias, as measured in a preliminary experiment (see S1 File for more details). There were eight repetitions per TestEI and video, resulting in a total of 144 trials (i.e., 8 repetitions * 9 TestEIs * 2 EIs).

Procedure

This experimental trial sequence, adapted from the one used by Courgeon et al. [23] and Thornton [24], is typical of representational momentum paradigms [3]. As illustrated in Fig 2, each trial started with a written message that remained on the screen for 2000 ms. This instruction invited the participants to blink and was aimed at preventing blinking during the trial itself. It was followed by a black screen, for a random duration of 800 to 1200 ms, and then by a fixation cross, for a random duration of 800 to 1200 ms. Randomization was used to prevent participants from anticipating the beginning of the videos. This was particularly crucial to avoid the build-up of a Contingent Negative Variation in the baseline (pre-stimulus) period, before the oncoming emotional event [41, 42]. A 500 ms static neutral expression was then displayed, followed by a dynamic expression of pain, i.e., a 117 ms video for the VideoEI50% condition and a 200 ms video for the VideoEI90% condition. The video was followed by a 267 ms mask (a pixelated face) and then a TestEI stimulus, which was displayed until each participant had responded. The participants had to estimate whether the TestEI was "equal to" or "different from" the final VideoEI. They had to answer by pressing the corresponding keyboard key (different = escape; equal = Enter). If reaction time to the TestEI exceeded 5 s, a "Wake up!" message was displayed and a beep signal sounded.

EEG recording and processing

All the experiments were conducted in the same lab and in exactly the same environmental conditions. EEG recordings were carried out using the BrainAmp system with active Ag-AgCl electrodes (ActiCap). Six of the twenty-five participants, (see 2.1 Participants section for details) wore a 32-electrode cap, whereas the remaining 19 participants wore a 64-electrode cap. The change in cap type came after the upgrading of our EEG system from 32 to 64 channels a few weeks after the beginning of our experiment. Both 32- and 64-electrode positions conformed to the 10–20 system. In order to avoid mixing data from 32 electrodes with data from 64 electrodes, only the channels corresponding to the 32-electrode cap for all analyses were kept. Recordings were made using the Brainvision Recorder at a 1000 Hz sample rate; no filter was applied during data acquisition. The FCz electrode was used as an electrical reference. All electrode impedances were no greater than 20 kΩ.

Brainvision Analyzer 2 was used to process data. Band pass filters were applied to EEG data (0.1–30 Hz, 12 dB/Octave). Then, data were segmented into periods that ranged from -1313 to 720 ms with respect to the TestEI display onset. Semi-automatic artifact rejection was used to remove data with artifacts: namely, a gradient greater than 50 μV/ms, a difference (maximum)

greater than an interval of 200 μV per 200 ms, and activity of less than an interval of 0.5 μV per 100 ms. Trials with ocular artifacts were also rejected during this process. The mismatch effect could manifest itself in the LPP component (i.e., up to 700 ms post-onset TestEI); the trials during which participants responded in less than 700 ms were therefore removed from our analysis. Trials were then baseline corrected, with a reference baseline taken between -1313 and -1213 ms pre-TestEI; that is to say, during the fixation point pre-video period. For each participant, data from the two video EIs were pooled in order to have enough data per congruity condition (i.e., TestEIs judged as congruent or incongruent with respect to the final video intensity). Finally, trials were averaged for each condition and participant. Note that the ERPs' time-values were corrected in order to consider the 13 ms latency of the display.

Data analysis

Behavioral data. We conducted two behavioral data analyses, with two complementary objectives: a) determined participants' individual PSE; and b) determined congruent and incongruent TestEI trials with respect to the participants' PSE, on an individual basis (see Fig 3 for an example). First, we computed the PSE values per VideoEI condition, for each participant, using a logistic probability density function fitted to the proportion of "equal" answers as a function of the TestEIs. The PSE provided an estimate of the TestEI stimuli, for which participants would answer "equal" with a probability of 1. The PSE was an estimation of the memorized intensity of the last frame of the video. With regard to statistical analysis, we conducted Student's *t* tests on the individuals' memory bias (see section Results for the results).

The second aim of this behavioral data analysis was to identify the trials which corresponded to TestEIs perceived as congruent or incongruent with respect to the memorized final EI (PSE) of each participant. This data would be used for the subsequent ERP analysis. In fact, our objective was to compare ERPs in response to the TestEIs which were perceived as equal to the memorized final EI (congruent TestEI) with ERPs in response to TestEIs that were perceived as different from the memorized final EI (incongruent TestEI). Therefore, the proportion of "equal" responses very seldom reached 1 for the TestEIs that were close to the PSE2, and symmetrically, did not always reach 0 for the TestEIs that were furthest from the PSE2. For this reason, on the basis of individual data, we identified the TestEIs that led to a proportion of "equal" responses superior to 60%, as "PSE congruent TestEIs" (see Fig 3: continuous line square). In contrast, we identified the TestEIs that had a proportion of "equal" responses inferior to 40%, as "PSE incongruent TestEIs" (Fig 3: dashed line square). In the example illustrated in Fig 3, the proportion of "equal" responses given to TestEI60% by the participants was approximately 0.9. Eight repetitions of TestEI60% stimulus were carried out; of these, a response of "different" was recorded only once. Thus, we did not keep this trial as a "PSE congruent TestEI". For the subsequent ERP analysis, we therefore only kept trials in which participants responded "equal" for the "PSE congruent TestEIs" and "different" for the "PSE incongruent TestEIs". Most importantly, for the ERP analysis, we set a minimum of 30 trials per studied condition.

After this, an average of 45.9 trials were available to each participant in the congruent condition. We kept 38.4 trials (15.82% of those rejected were due to artifacts and < 700 ms responses). Similarly, of the 61 trials available on average in the incongruent condition after applying the initial behavioral criterion, we kept 47.4 trials per participant (20.4% of those rejected were due to artifacts and < 700 ms responses). Although we had more incongruent elementary trials (n = 61) than congruent trials (n = 45.9) for the ERP analysis ($t(24) = 3.72$, $p = .001$), the rejection rates due to artifacts and < 700 ms responses did not significantly differ between the two conditions ($t(24) = 1.85$, $p = .08$).

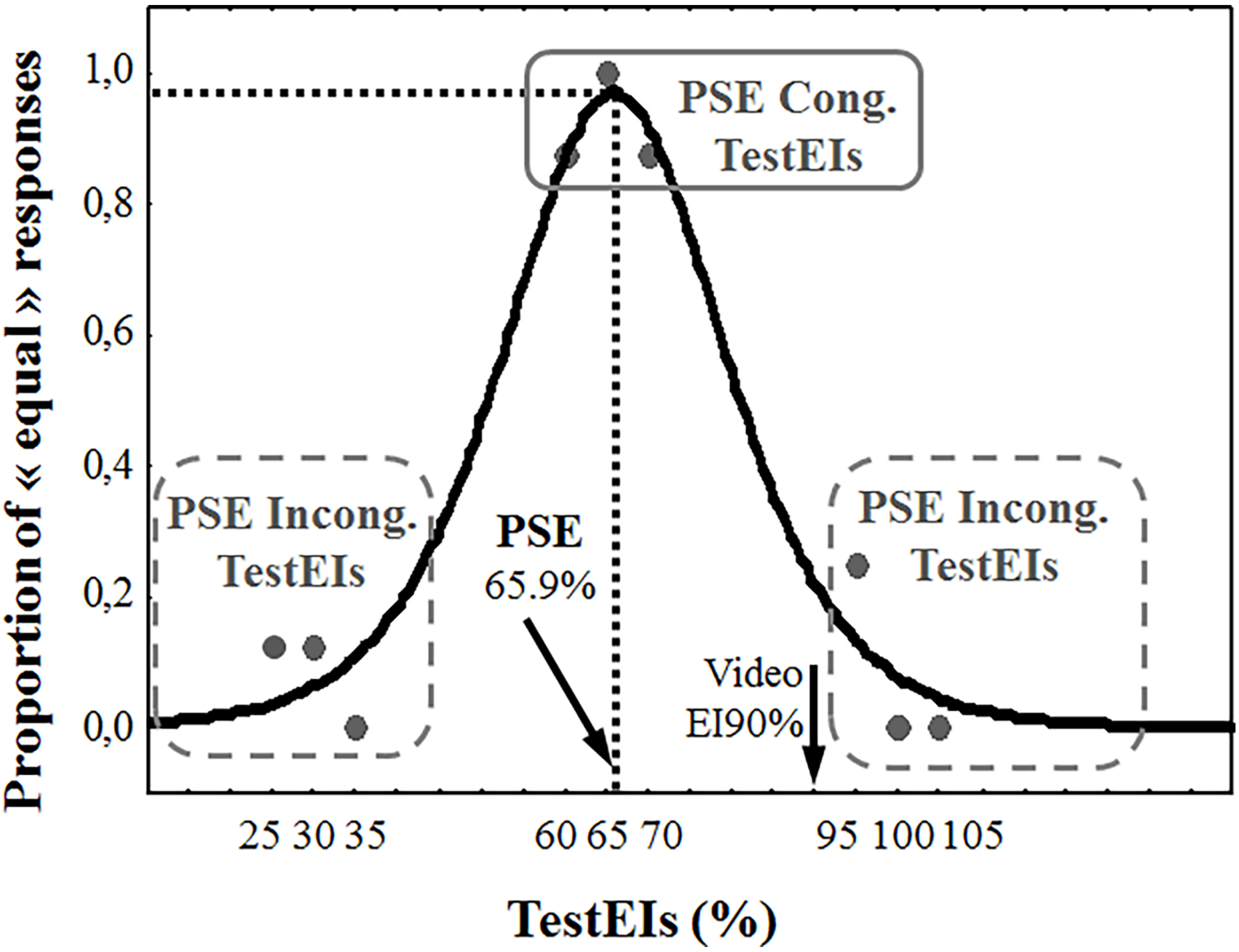

Fig 3. Example of behavioral results from one participant, in response to the VideoEI90% condition. Participants presented a negative memory bias, with a PSE of 65.9%, for a video stopping at 90% of the maximal intensity. Triplets of TestEIs are represented by gray dots.

https://doi.org/10.1371/journal.pone.0200535.g003

Existing psychophysics literature has shown greater RTs in response to stimuli close to PSEs [43]. With regard to the mismatch effect, in order to confirm that incongruent stimuli were easier to detect, we tested whether reaction time to congruent trials was greater than to incongruent trials (see section Results for results).

Event-related potentials (ERPs). Data from EEG electrodes were grouped into four functional anatomical regions of interest (ROIs): frontal (F), centro-parietal (CP), temporal (T) and occipital (O), as illustrated in Fig 4. The occipital and temporal regions are known to be associated with the P100 and N170 ERPs, concomitant with visual (and especially face) processing [44]. With regard to the centro-parietal and frontal regions, they are supposed to be sensitive to higher level processes such as context integration (reflected by the N400 ERP [45]). ERP post-onset TestEI data were analyzed using the averaged signal amplitude of three subsequent but non-overlapping time windows: [50–140 [ms for the P100 ERP; [140–330 [ms for the N170 ERP; and [330–700] ms for the LPP ERP. Indeed, instead of performing a point by point analysis in the time domain or analyzing ERP peaks, we performed a time-window analysis which consisted in dividing waveforms into a series of time windows (see [46]). Accordingly, in our statistical analyses, there was only one data point per time window and for each

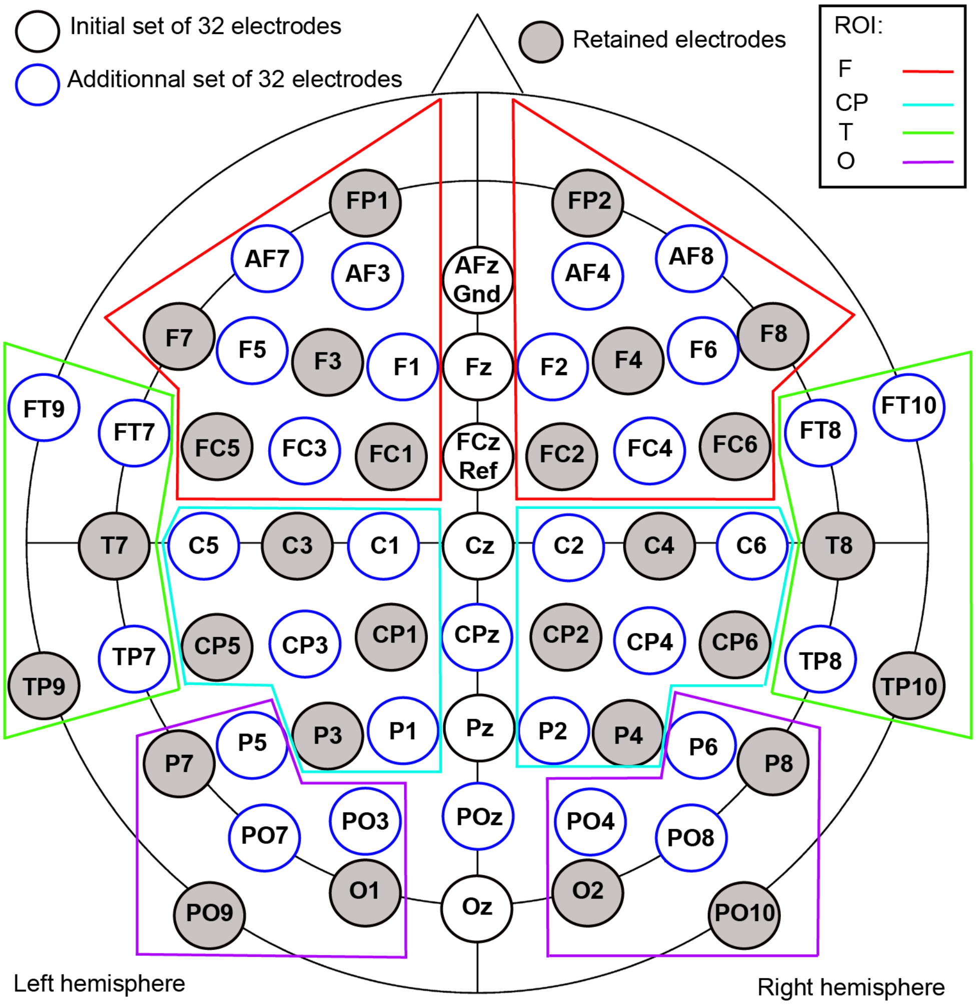

Fig 4. Electrode array. The data from six participants were recorded using 32 electrodes, whilst the data from the remaining 19 participants were recorded using 64 electrodes. For the purposes of statistical analysis, we grouped electrodes into four ROIs, based on anatomical and functional criteria: Frontal (F), Centro-Parietal (CP), Temporal (T) and Occipital (O).

https://doi.org/10.1371/journal.pone.0200535.g004

"congruity x ROI x hemisphere" cell. This point corresponded to the average signal in a given time window and condition.

Source localization analysis was performed for each brain wave component using the LORETA transform function of BrainAnalyzer (Low Resolution Electromagnetic Tomography [47]) which computes the full three-dimensional current density distribution (the current density field). Through this analysis, it was possible to estimate the neurocognitive sources of the previously identified ERPs.

The modulation of ERP by congruity (between TestEI and memorized expression intensity) was examined using both MANOVAs (multivariate analyses of variance) and ANOVAs (univariate analyses of variance) on the mean amplitude value (μV), as recommended by [48] for testing repeated measure effects in psychophysiological data. We provide MANOVA results together with Wilks' lambda (*Wilks'* Λ), and ANOVA results with η^2_p (partial eta squared). *Wilks'* Λ is the multivariate counterpart of $1—\eta^2$ for univariate variables (see [49]). It provides a measure of the proportion of variance in the combination of dependent variables

unaccounted for by a factor. In contrast, η^2 is a measure of effect size in terms of proportion of variance explained by a factor. The *p* values for ANOVAs were corrected, when appropriate, using the Greenhouse-Geisser correction, as recommended by [50]. Post-hoc pairwise comparisons were assessed by means of planned comparisons with a significance threshold corrected with the use of the Bonferroni criterion (α = .05 / n comparisons). Furthermore, *p* values were considered as significant up to .05 and marginally significant between .05 and .10. Significant or marginally significant differences in the amplitude of the ERPs between the incongruent and the congruent condition were termed the "mismatch effect". Only significant or marginally significant effects would be reported.

Finally, mediation models of the mismatch effect were tested with path analysis using PROCESS v2.13, a macro created by [51] for SPSS. Bias-corrected bootstrap 95% confidence intervals were constructed with 10,000 samples (i.e., bootstrap estimates of the indirect effect) for testing the indirect effects. Confidence intervals that do not contain zero support the conclusion that the indirect effect has reached a "statistically significant" degree (see [51], 2013, p.109). In contrast, according to [51], (p.158): "The fact that a confidence interval for an effect contains zero does not mean the effect is zero. It merely means that zero is in the realm of possibility, or that one cannot say with certainty what the direction of the effect is." Bootstrap confidence intervals (BootCI) will be provided between parentheses.

Results

Behavioral data

We found a significant negative memory bias (i.e., a bias significantly different from 0) in the VideoEI50% condition (-8.6%, SD = 17.2%, ranging from -45.4% to +18.9%), $t(24) = 2.5$, $p = .02$, and in the VideoEI90% condition (-20.4%, SD = 21.3%, ranging from -75.2% to +3.7%), $t(24) = 4.7$, $p = .00009$. Memory displacement (bias values) varied with VideoEI conditions (50% and 90%), $t(24) = 5.1$, $p = .00003$. Variances of bias values among both VideoEI conditions were not significantly different (Fisher's test, $F(1, 24) = .65$, $Var_{VideoEI50\%} = 307.2$, $Var_{VideoEI90\%} = 471.8$, $p = .15$). This analysis allowed us to average the two VideoEI conditions in the ERP analysis of the mismatch effect.

Further analysis of the behavioral data was conducted using reaction time data in order to test our hypothesis that incongruent stimuli are more easily detected than congruent stimuli (see the end of the Behavioral data subsection, in the Data analysis section). An ANOVA with two factors (VideoEI × congruity) confirmed our hypothesis, with a significant main mismatch effect on participants' reaction times, $F(1, 24) = 8.7$, $p = .007$, ($M_{congruent} = 1263.5$ ms, $M_{incongruent} = 1180.0$ ms), together with no significant effect of VideoEI, and no significant interaction between VideoEI and congruity.

Event-related potentials (ERP)

Figs 5 and 6 illustrate the ERP signal data for the entire trial sequence, from the baseline to the post-onset TestEI. Statistical analyses were conducted only on ERPs for the TestEI stimuli.

Three ERP components were evoked by TestEI onsets. We identified the first component as a P100 wave, peaking in the O ROI, around 100 ms after the stimulus onset. We only found a marginal mismatch effect on this wave (details are presented hereafter). The second component was a N170 wave, peaking in the O ROI around 230 ms after face onset. This wave presented a greater deflection in the right hemisphere than in the left.

Lastly, we identified an LPP wave, peaking around 500 ms post TestEI onset. The amplitude of this wave was left lateralized in the T ROI. The LPP deflection was also reduced for incongruent trials in all ROIs. LORETAs, presented in Fig 7, illustrate the posterior-lateral

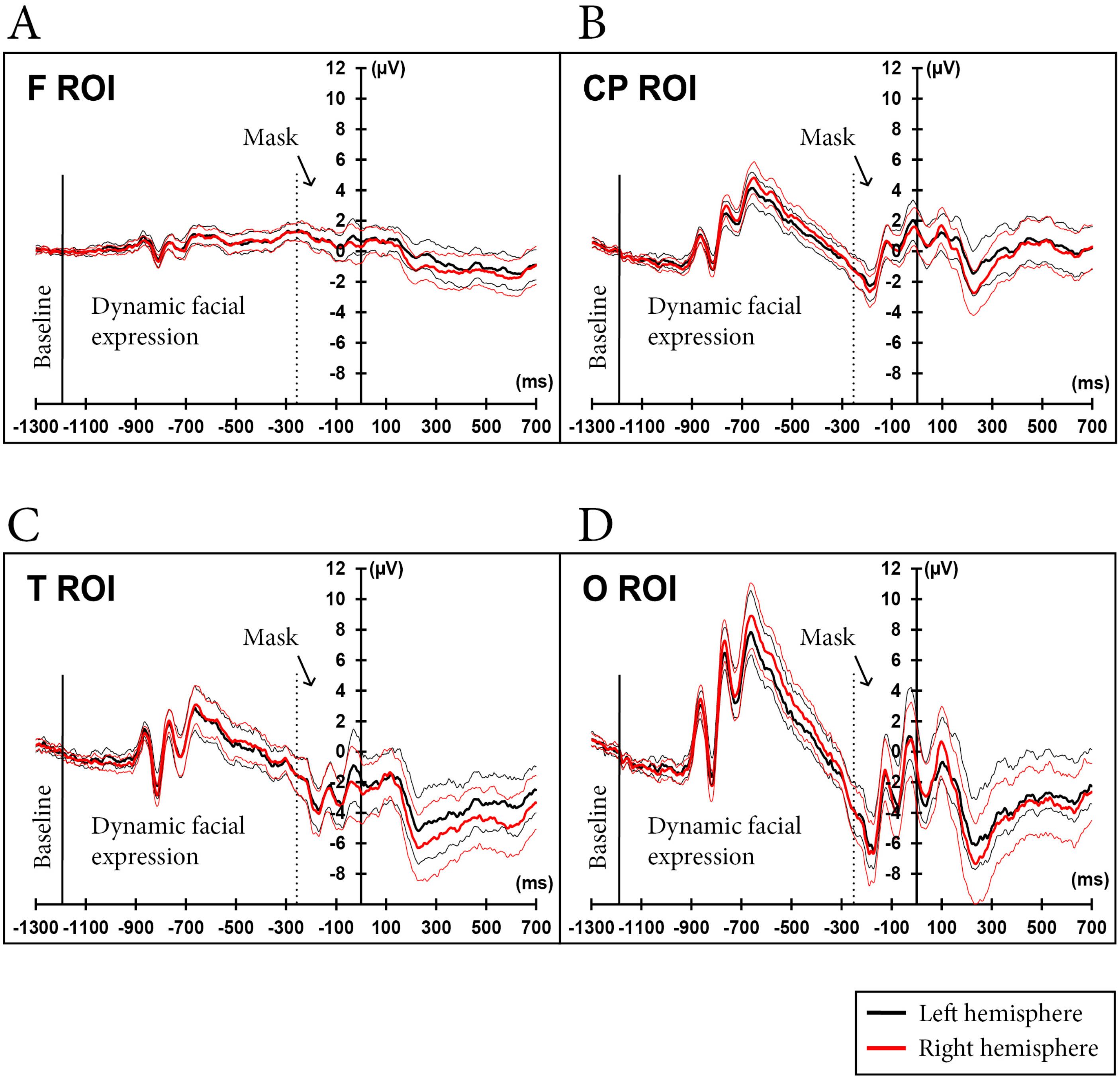

Fig 5. Average ERPs, by hemisphere (with congruity conditions combined). Apart from the baseline, data recorded before the 0 ms time value (i.e., data recorded during the presentation of the dynamic facial expression and the mask) are presented for illustrative purposes only. In the T ROI (panel C), the average amplitude of the EEG signal (across all time windows) was more negative in the right hemisphere (M = -3.81 μV) than in the left hemisphere (M = -2.98 μV). In the O ROI, significantly greater deflections were observed in the right hemisphere than in the left hemisphere, for both P100 and N170 waves (panel D).

https://doi.org/10.1371/journal.pone.0200535.g005

distribution of the neural generators of the three waves, with a tendency to be distributed more anteriorly for the mismatch effect at the LPP wave.

Multivariate and univariate analyses. An initial MANOVA was performed in which mean μV values extracted from each ROI (Frontal, Centro-Parietal, Temporal, and Occipital)

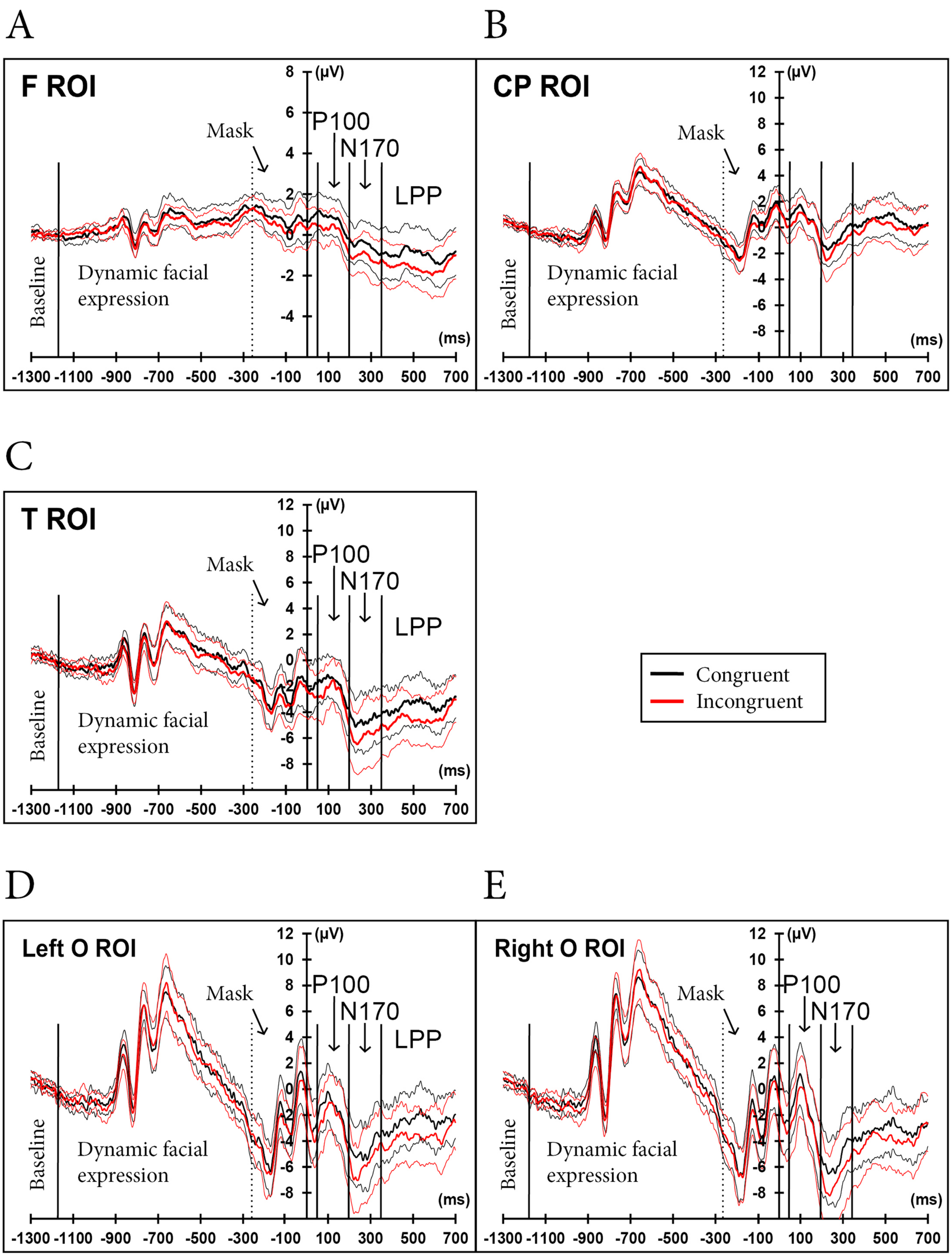
A
F ROI
B
CP ROI
C
T ROI
D
Left O ROI
E
Right O ROI
Mask
P100
N170
LPP
Baseline
Dynamic facial expression
(μV)
(ms)
-1300 -1100 -900 -700 -500 -300 -100 100 300 500 700
Congruent
Incongruent

Fig 6. Average ERPs, by congruity condition (with hemispheres combined). Apart from the baseline, data recorded before the 0 ms time value (i.e., data recorded during the presentation of the dynamic facial expression and the mask) are presented for illustrative purposes only. Taking all time windows together, the mismatch effect was significant in the T (panel C) and O (panels D and E) ROIs. It was also marginally significant in the F ROI (panel A). In contrast, the mismatch effect in the O ROI varied across time windows. It showed marginally weaker P100 amplitude in the incongruent condition than in the congruent condition, and marginally greater N170 amplitude in the incongruent condition than in the congruent condition. However, the difference in the mismatch effect on the LPP component was significant in the left O ROI and not in the right O ROI.

https://doi.org/10.1371/journal.pone.0200535.g006

were considered as different dependent variables, with a time window (P100, N170, LPP), congruity (2), and a hemisphere (2 for each ROI) as within-subject factors. Along the lines of [46], instead of conducting a series of independent tests, we included time windows as a factor in a single statistical test. Note that the size of each time window does not need to be constant (see [46], p.48).

The MANOVA results are summarized in Table 1. The main, interesting MANOVA effect, handling ROIs altogether simultaneously, was that the mismatch effect varied with the time window and brain hemisphere ($p < .005$, see Table 1). Subsequent MANOVA follow-up results handling ROIs separately, showed a mismatch effect in the temporal and occipital regions (*ps* $< .05$), and marginally in the frontal region ($p < .079$). In addition, the results evidenced that the mismatch effect varied with the time window and brain hemisphere in the occipital region ($p < .01$). Consequently, follow-up ANOVAs were conducted on data from the O ROI only, for each time window (P100, N170, LPP) separately, with congruity (2), and hemisphere (2 for each ROI) as within-subject factors.

The ANOVA on the P100 time window in the O ROI showed a marginal mismatch effect, $F(1, 24) = 3.50$, $p = .07$, $\eta^2_p = .13$, reflecting a greater deflection in response to congruent TestEIs ($M_{congruent}$ = -.70 μV; $M_{incongruent}$ = -1.48 μV). A significantly greater overall positive deflection in the right hemisphere was also evidenced, $F(1, 24) = 4.78$, $p = .039$, $\eta^2_p = .17$, ($M_{Left\ hemisphere}$ = -1.45 μV; $M_{Right\ hemisphere}$ = -.73 μV). However, the mismatch effect did not vary with the hemisphere, $F(1, 24) < 1$, n.s.).

The ANOVA on the N170 time window in the O ROI showed a marginal mismatch effect, $F(1,24) = 4.16$, $p = .05$, $\eta^2_p = .15$, reflecting a greater deflection in response to incongruent TestEIs ($M_{congruent}$ = -4.53 μV; $M_{incongruent}$ = -5.55 μV). A significantly greater overall negative deflection in the right hemisphere was also evidenced, $F(1, 24) = 5.99$, $p = .022$, $\eta^2_p = .20$, ($M_{left\ hemisphere}$ = -4.60 μV; $M_{right\ hemisphere}$ = -5.48 μV). However, the mismatch effect did not vary with the hemisphere, $F(1, 24) < 1$, n.s.).

Finally, the ANOVA on the LPP time window in the O ROI showed a marginal mismatch effect, $F(1,24) = 4.11$, $p = .05$, $\eta^2_p = .15$, reflecting a greater deflection in response to congruent TestEIs ($M_{congruent}$ = -2.90 μV; $M_{incongruent}$ = -3.83 μV). No effect of the brain hemisphere was evidence, $F(1, 24) = 1.60$, $p > .21$. However, the mismatch effect varied significantly with the brain hemisphere, $F(1, 24) = 4.70$, $p = .04$, $\eta^2_p = .16$. Bonferroni-corrected planned comparisons showed a mismatch effect in the left hemisphere with a greater deflection in response to congruent TestEIs ($M_{congruent}$ = -2.59 μV; $M_{incongruent}$ = -3.74 μV), $F(1, 24) = 6.98$, $p = .014$ ($p < .05/2$), but not in the right hemisphere, $F(1, 24) = 2.06$, $p > .16$.

Individual differences. The distribution of the mismatch effect across the participants for the different ERP components (P100, N170, and LPP) can be visually appreciated via a scatterplot of the mean amplitude values of the incongruent trials over those of the congruent trials (see Fig 8), along the lines of [52]. Here, we only examined individual differences in the ROIs showing a marginal or significant mismatch effect according to the MANOVA. We considered that when the paired observations (one dot per participant) fell on the unity line ± .50 μV, no mismatch effect was shown. Although .50 μV is an arbitrary criterion; our idea was to define values that could not be truly differentiated from 0. Note that this criterion is consistent with

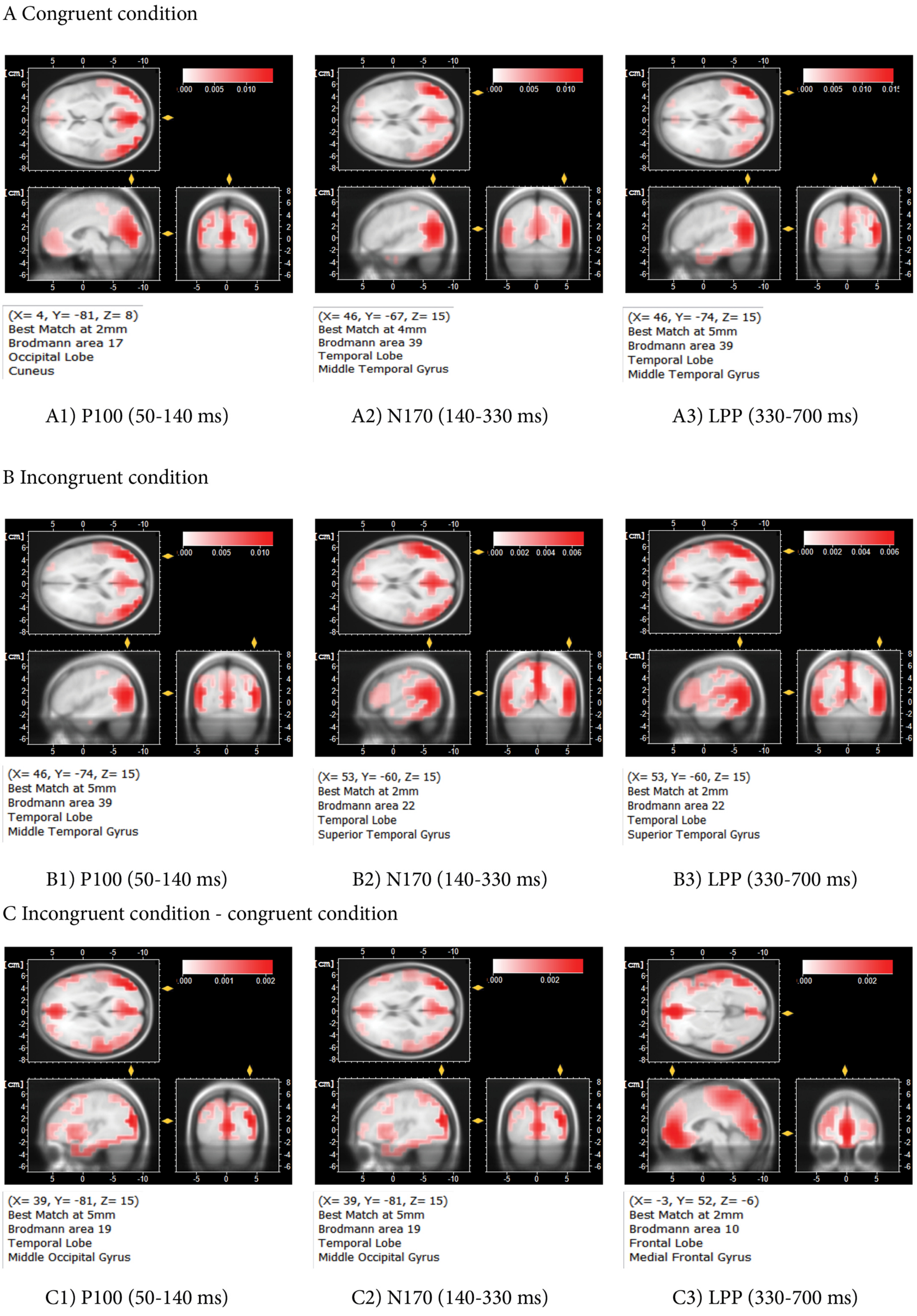
A Congruent condition
(X= 4, Y= -81, Z= 8)
Best Match at 2mm
Brodmann area 17
Occipital Lobe
Cuneus
(X= 46, Y= -67, Z= 15)
Best Match at 4mm
Brodmann area 39
Temporal Lobe
Middle Temporal Gyrus
(X= 46, Y= -74, Z= 15)
Best Match at 5mm
Brodmann area 39
Temporal Lobe
Middle Temporal Gyrus
A1) P100 (50-140 ms)
A2) N170 (140-330 ms)
A3) LPP (330-700 ms)
B Incongruent condition
(X= 46, Y= -74, Z= 15)
Best Match at 5mm
Brodmann area 39
Temporal Lobe
Middle Temporal Gyrus
(X= 53, Y= -60, Z= 15)
Best Match at 2mm
Brodmann area 22
Temporal Lobe
Superior Temporal Gyrus
(X= 53, Y= -60, Z= 15)
Best Match at 2mm
Brodmann area 22
Temporal Lobe
Superior Temporal Gyrus
B1) P100 (50-140 ms)
B2) N170 (140-330 ms)
B3) LPP (330-700 ms)
C Incongruent condition - congruent condition
(X= 39, Y= -81, Z= 15)
Best Match at 5mm
Brodmann area 19
Temporal Lobe
Middle Occipital Gyrus
(X= 39, Y= -81, Z= 15)
Best Match at 5mm
Brodmann area 19
Temporal Lobe
Middle Occipital Gyrus
(X= -3, Y= 52, Z= -6)
Best Match at 2mm
Brodmann area 10
Frontal Lobe
Medial Frontal Gyrus
C1) P100 (50-140 ms)
C2) N170 (140-330 ms)
C3) LPP (330-700 ms)

Fig 7. Source localizations (LORETA) for each congruity condition and the mismatch effect, per time window. The LORETA suggested that the main source of the P100 wave was in the occipital lobe in the congruent condition (A1) and in the posterior temporal lobe in the incongruent condition (B1), with the source of the mismatch effect located in the posterior temporal lobe (C1). The sources of the N170 and LPP waves were mainly localized in the posterior temporal lobe in both the congruent condition (A2 and A3) and the incongruent condition (B2 and B3). The strongest sources of the N170 and LPP mismatch effect were in the temporal lobe (C2 and C3), and the frontal lobe, respectively.

https://doi.org/10.1371/journal.pone.0200535.g007

Table 1. Multivariate tests. Significant effects are highlighted in bold.

Effect	*Wilks' Λ* value	Hypothesis df	Error df	*F*	*p* value
**Time window (A)**	**0.18**	**8**	**17**	**9.58**	**<0.0001**
Congruity (B)	0.77	4	21	1.57	0.22
Hemisphere (C)	0.82	4	21	1.18	0.35
A × B	0.83	8	17	0.44	0.88
**A × C**	**0.20**	**8**	**17**	**8.25**	**<0.001**
B × C	0.83	4	21	1.08	0.39
**A× B× C**	**0.29**	**8**	**17**	**5.15**	**<0.005**
Frontal ROI					
**Time window (A)**	**0.40**	**2**	**23**	**17.20**	**<0.0001**
Congruity (B)	0.88	1	24	3.37	0.08
Hemisphere (C)	0.93	1	24	1.89	0.18
A × B	1.00	2	23	0.00	1.00
**A × C**	**0.52**	**2**	**23**	**10.46**	**<0.001**
B × C	0.96	1	24	1.10	0.30
A× B× C	0.96	2	23	0.52	0.60
Centro-parietal ROI					
**Time window (A)**	**0.43**	**2**	**23**	**14.99**	**<0.0001**
Congruity (B)	0.92	1	24	2.02	0.17
Hemisphere (C)	0.94	1	24	1.66	0.21
A × B	1.00	2	23	0.04	0.96
**A × C**	**0.32**	**2**	**23**	**24.39**	**<0.00001**
B × C	0.97	1	24	0.75	0.39
A× B× C	0.88	2	23	1.54	0.24
Temporal ROI					
**Time window (A)**	**0.48**	**2**	**23**	**12.45**	**<0.001**
**Congruity (B)**	**0.84**	**1**	**24**	**4.66**	**<0.05**
**Hemisphere (C)**	**0.83**	**1**	**24**	**4.91**	**<0.05**
A × B	0.97	2	23	0.30	0.74
**A × C**	**0.66**	**2**	**23**	**6.02**	**0.01**
B × C	0.96	1	24	0.95	0.34
A× B× C	0.98	2	23	0.19	0.83
Occipital ROI					
**Time window (A)**	**0.43**	**2**	**23**	**15.40**	**<0.0001**
**Congruity (B)**	**0.81**	**1**	**24**	**5.50**	**<0.05**
Hemisphere (C)	0.98	1	24	0.41	0.53
A × B	0.97	2	23	0.30	0.75
**A × C**	**0.45**	**2**	**23**	**13.78**	**<0.001**
B × C	0.99	1	24	0.35	0.56
**A× B× C**	**0.64**	**2**	**23**	**6.49**	**<0.01**

https://doi.org/10.1371/journal.pone.0200535.t001

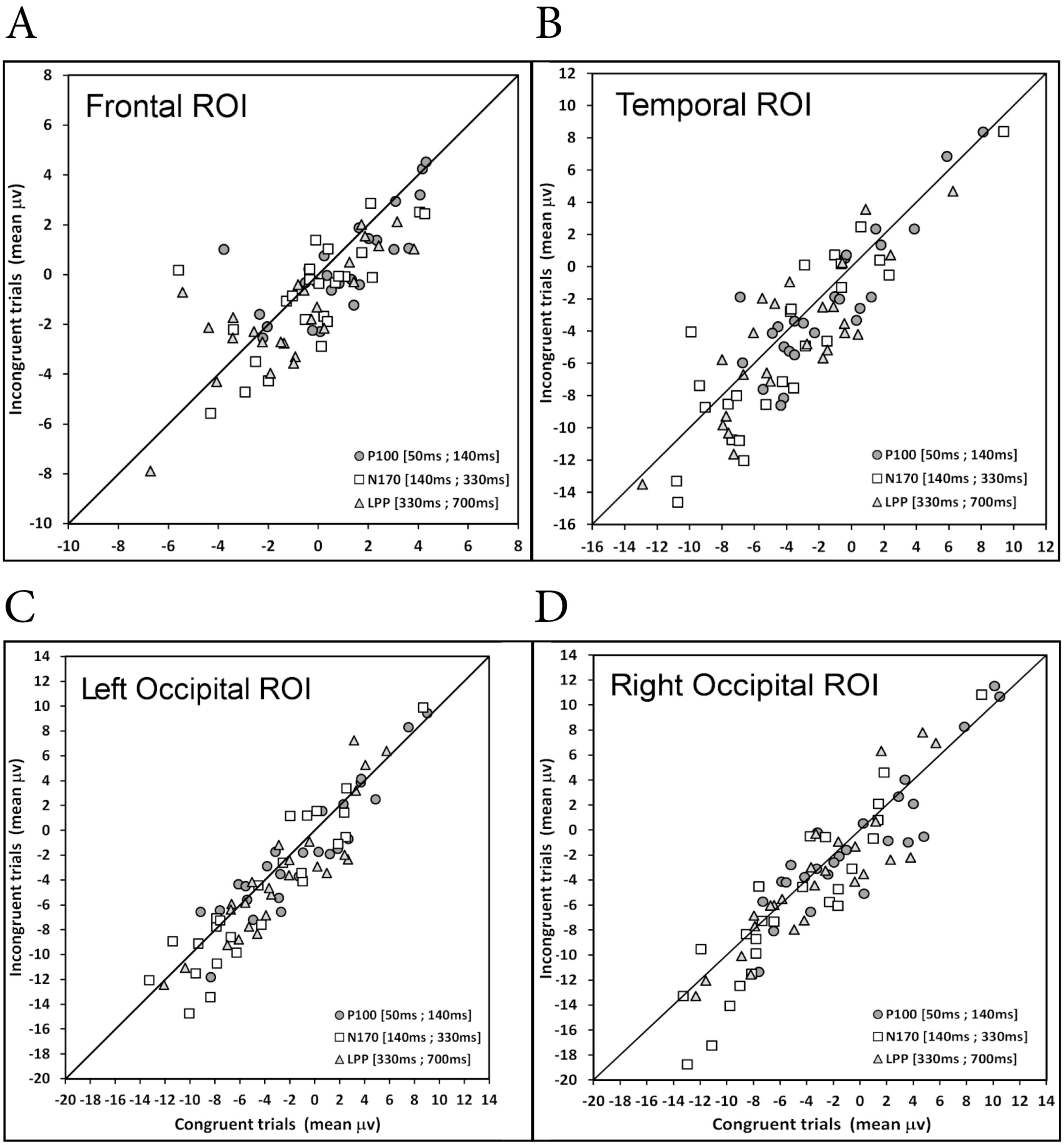

Fig 8. Scatterplots of the distribution of the mismatch effect (A) and of the hemisphere effect (B) across the participants, for the F ROI and T ROI, as well as each hemisphere of the O ROI, for each ERP component (P100, N170, and LPP).

https://doi.org/10.1371/journal.pone.0200535.g008

the mean marginal or significant mismatch effects in the Frontal, Temporal and Occipital ROIs that were greater than .50 μV, whereas the absence of mismatch effect in the CP ROI corresponded to mean effects below or equal to .50 μV.

Let's first consider the F ROI and T ROI that showed an overall mismatch effect across the three ERP components. For the F ROI, the number of participants showing no effect was 9 for the P100, 4 for the N170, and 7 for the LPP, that is to say, 26.7% of the participants on average. For the T ROI, there were few (5.3%), with only 1 or 2 participants depending on the time window. In contrast, the majority of paired observations were below the unity line for the F ROI (54.7%) and the T ROI (64%), demonstrating an overall mismatch effect with little variation (SD = 5%) across the time windows. The participants localized above the unity line were 18.7% and 30.7%, for the F ROI and R ROI, respectively. Given that idiosyncratic factors such as personality traits may reverse the direction of effects (see [53] for an example of the effect of empathy on the N400), if we examine the paired observations that fell outside the unity line (i.e., > |.50| μV), it corresponded to 73.4% and 94.7% of the participants for the F ROI and R ROI, respectively. With regard to the occipital region, the number of participants showing no mismatch effect (< |.50| μV) totaled 20% (n = 5 in each time window) for the Left O ROI, and 18.7% (4 or 6 depending on the time window) for the Right O ROI. The majority of paired observations were below the unity line for the O ROI in the left (50.7%) and right ROI (52%) hemisphere, with a smaller dispersion in the left hemisphere (SD = 1.2μV) than the right hemisphere (SD = 1.9 μV), as illustrated in Fig 8. The number of participants localized above the unity line totaled approximately 29% in each occipital ROI.

Path analysis. To investigate the temporal unfolding of the mismatch effect from Occipital P100 to Left Occipital LPP, we adopted a path analytic approach along the lines of [51]. Path analysis using mediation models has already been applied to EEG data analysis and face perception [54], as well as to fMRI data showing that the influence of the amygdala on the processing of visual stimuli, such as the face, is both direct (on the visual cortices) and indirect (via the frontal region) [55]. Details on the different theoretical assumptions guiding our own path analytic analyses, as well as the interpretation with respect to our LORETA findings, are available in the *General Discussion*.

Our path analysis mediation models were tested on the basis of the individual mismatch effect data (mean μV difference between incongruent and congruent trials) illustrated in Fig 8. We only considered the data of F ROI, T ROI, and O ROI because they showed either marginal or significant mismatch effects in the MANOVA. Moreover, in order to simplify the models and make them testable with path analysis, we considered the mismatch effect at Occipital P100 (O_P100) as the initial input, and the mismatch effect at Left Occipital LPP (Left_O_LPP) as the dependent variable to be predicted. Three path analytic models were tested: a) an initial serial multiple mediator model (see [51] p.143 and after) with two mediators, namely the mismatch effect at Frontal N170 and Temporal N170; b) a model with two simple mediation models arranged in series; and c) a serial multiple mediator model with three mediators. For the sake of simplicity, only the third model will be described here. The two initial models are described in S2 File.

The model illustrated in Fig 9 is a serial multiple mediator model with three mediators: the mismatch effect at Frontal N170 (F_N170), Temporal (N170), and at Frontal LPP (F_LPP). It models the unfolding of the mismatch effect from O P100 to Left O LPP. The results evidenced a significant "O_P100 ➔ F_N170 ➔ F_LPP ➔ Left_O_LPP" indirect effect (BootCI = [.0474; 1.082]), concomitant with a Direct effect of O_P100 on Left_O_LPP, $p = .022$. This indirect effect (see Fig 9) suggests that the Frontal region (possibly the Medial Frontal Gyrus) plays a crucial role in mediating the mismatch effect observed in the Left Occipital ROI at the LPP time-window.

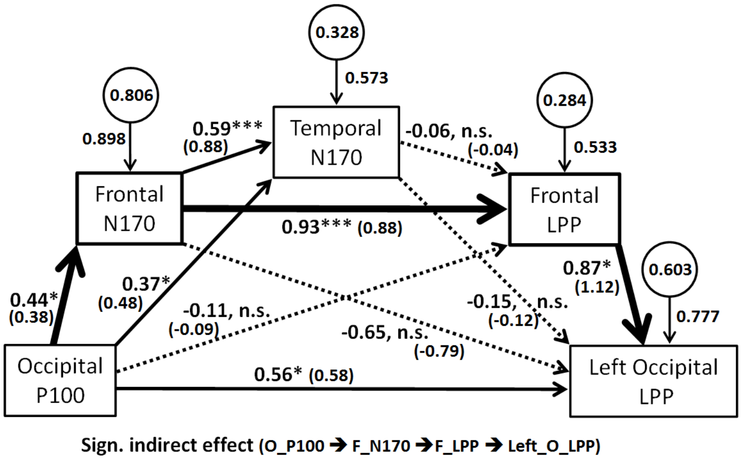

Fig 9. Path analysis mediation model testing for the spatio-temporal unfolding of the mismatch effect from occipital P100 up to Left occipital LPP. This serial multiple mediator model includes three mediators (F_N170, T_N170, and F_LPP). Standardized (β) coefficients (in bold) are provided together with unstandardized coefficients (between parentheses). The unstandardized coefficients are in μV unit, together with the significance level of effects as §$p < .10$, $^{*}p < .05$, $^{**}\ p < .01$, $^{***}\ p < .001$. Significant indirect effects are illustrated with thicker lines.

https://doi.org/10.1371/journal.pone.0200535.g009

Discussion

The present study investigated the neurocognitive mechanisms involved in the emotional anticipation of facial expressions of pain with the help of behavioral measures and ERPs. Using a representational momentum paradigm, we measured, in the first place, the emotional anticipation (memory displacement) of facial pain expressions. In the second place, we measured ERPs in response to static facial intensities, which were perceived as congruent or incongruent with respect to the memorized intensity.

Individuals always attempt to anticipate and predict events. At a representational level, Miceli and Castelfranchi [56] have suggested that our emotions often reflect our anticipation of forthcoming events, with dialectical interaction between "what is" and "what is not (yet)". Thus, the anticipation of future events can induce an emotion (e.g., fear, hope, and trust), and the confirmation, or not, of this anticipation can elicit a reaction in the form of another emotion (e.g., surprise, discouragement, and regret). The anticipation process can be studied at the representational level (for a review, see [56]), but also at a more perceptual lower level. In fact, these expectancies can lead to the emotional anticipation of a change in facial expression [25], which may induce memory bias [4, 23, 24]. When an expression is not congruent with an individual's anticipated expression, either in nature (i.e., emotional valence) or in intensity, our trust in others may be challenged.

In the present study, we focused on emotional anticipation which accompanies the perception of facial expressions of pain. In truth, the ability to anticipate and thereby rapidly detect the presence of an expression of pain seem to have both a social importance and an adaptive function [8, 9]. We used a representational momentum paradigm inspired by Courgeon et al. [23] and Thornton [24], to explore emotional anticipation in response to facial expressions, combining behavioral and neurocognitive (ERP) measures. The participants had to compare the intensity of a static test expression with the memorized final intensity of a previously displayed dynamic expression. We theorized that a mismatch effect may be considered as a neurocognitive biomarker of anticipatory processes for facial expressions. We anticipated that ERPs would be modulated by a TestEI which deviates from the memorized expression intensity (i.e., incongruent trials). However, the idiosyncratic nature of memory biases meant that

incongruent and congruent trials could only be defined on an individual basis. Therefore, in order to consider individual variability in emotional anticipation, we adapted TestEI stimuli for each participant as a function of his or her memory bias, as previously measured in a preliminary experiment. To our knowledge, this participant-centered approach has never been proposed until now.

Assuming that emotional anticipation accompanies our perception of a dynamic event such as an emotional facial expression, if the stimulus abruptly disappears, four scenarios may be considered. The observer may consider that: a) the emotional facial expression can be maintained at its final intensity for a period of time (e.g., apex duration can vary with pain context, see [57, 58]); b) the expression intensity may increase; c) the facial expression will once again become neutral; or d) there may be a qualitative change (e.g., from surprise to joy). Scenarios "a" to "c" reflect participants' internal representations of the dynamic progression of a facial expression, which is commonly composed of three stages: onset (increasing), apex (maximum) and offset (decreasing). As a consequence, if a memory displacement was to occur during the mask stimulus displayed before the TestEI, this memory bias would be null, positive, or negative, respectively, for cases a, b, and c. Here, we do not consider scenario "d", given that the participants only observed facial expressions of pain. We found that memory bias varied both with the final expression intensity to be memorized (whether 50% or 90% VideoEI) and at the inter-individual level.

The overall negative memory bias was smaller for the facial expression of medium intensity (VideoEI50%) than for the facial expression of high intensity (VideoEI90%). However, in terms of the proportion of the displayed dynamic emotional intensity, the bias was roughly equivalent (approximately 20% of the final intensity). Thus, the overall negative memory bias is consistent with the expectation of a return to a neutral expression. Although this negative memory bias concords with findings in previous studies [23, 24], literature has also shown positive memory bias for facial expressions [4]. The added value of our study with respect to this literature is based on the fact that we considered the importance of inter-individual variability of memory bias (negative, positive, or null depending on the participant) to investigate any underlying neurocognitive processes.

Studies that have examined simulation theory and the embodiment of other people's facial expressions [27, 59] have suggested that an involuntary motor simulation of the observed facial expression causes the observer to "experience" the observed action, which in turn gives us information about the agent's emotional state. Previous studies have supported the idea of a dynamic facial expression (displayed before the mask), which induces an immediate perceptual history (here, expression intensity progresses from 50% or 90%) among participants before "emotional anticipation" [6, 25]. Contrary to physical events which induce forward memory displacement (i.e., a representational momentum effect), biological motion may induce memory displacement in different directions (see [3, 60] for reviews). Thus, we consider the term "emotional anticipation" more appropriate with regard to facial expressions of pain.

According to literature on event perception and cognition (e.g., [5, 61]), the observed motion in daily life may temporarily disappear, or the scene may change dramatically because of a perceptive change (such as a camera cut during filming). Nonetheless, information is still available to the perceiver on the basis of his or her representation of the event. When a moving or changing stimulus reappears, we can easily detect if the current state of the event has deviated from what is expected. Although our participants were instructed to memorize rather than extrapolate the final expression intensity of a facial expression of pain, emotional anticipation may well be irrepressible [62, 63]. Accordingly, our participants were able to detect more rapidly a test expression whose intensity had deviated from the expected expression

intensity (whatever the memory displacement). Taken together, all neuroelectric markers (P100, N170 and LPP) were concomitant with this mismatch effect in the temporal and occipital regions, and, marginally, in the frontal region. Unexpected intensities of the test expression of pain decreased the P100 amplitude and increased the N170 deflection. These findings suggest an influence of high-level expectancies on low-level rapid perceptual processes, with expectancies acting as priming representations for forthcoming emotional expressions [64]. The latency of the N170 peak (peaks not studied but visible in Figs 5 and 6) was closer to 250 ms than 170 ms after TestEI onset. A possible explanation would be that the N170 component could be merged with an N250r component. In effect, the N250r wave was shown to exist for repeated presentations of faces [65–68]. Indeed, we presented multiple repetitions of the same face.

We also examined mismatch effects at the decision-making processing stage. According to Ibanez et al. [44], the LPP is a late positive component modulated by either the motivational relevance or semantic valence of the stimulus, and by contextual information. We found that unexpected emotional expression intensities led to lower LPP wave amplitudes, compared with expected intensities. This LPP modulation may be interpreted in view of behavioral response times. The higher amplitude of LPP and greater response time for expected pain intensities are consistent with typical psychophysical data that show greater RTs in response to stimuli that are close to PSEs [43]. We propose that the P100 and N170 waves reflect the comparison process between the expected (memorized) and test emotional intensities at an early stage of visual processing. In contrast, the LPP wave might reflect a verification process at a higher cognitive level. Indeed, Ibanez et al. [44] reported that a similar component, the LPC (Late Positive Component), also reflects such a verification process. In the occipital region, the mismatch effect was only significant in the left hemisphere. We found a study [69] in which the authors also described a (marginally significant) effect on the LPP wave in the left occipital hemisphere but not in the right. The study addressed the categorization of angry versus neutral faces by children. However, the authors did not specifically refer to the effect this had on the left occipital cortex. It might be interesting to investigate at greater length the specific role of the left occipital hemisphere in the late processing of emotional stimuli.

LORETA source localization showed the activation of different cortical areas involved in facial processing in response to emotional test intensities (cf. Fig 9). The neural sources of the P100 wave appeared to be mainly located in the Cuneus (occipital lobe, BA 17) for the congruent condition and in the pMTG (the posterior part of Middle Temporal Gyrus, BA 39) for the incongruent condition. The greater activation mismatch effect in the P100 time window was located in the MOG (Middle Occipital Gyrus, BA 19). With regard to the N170 wave, neural sources were stronger in the pMTG for the congruent condition, and in the pSTG (the posterior part of Superior Temporal Gyrus, BA 22) for the incongruent condition. The neural sources of the mismatch effect on the N170 wave were mainly located in the MOG (BA 19). pMTG and pSTG are the visual entry point of a bottom-up activation of the mirror neuron system [70]. pSTG contributes to a neural network which subserves face-based mentalization, by extracting information about intentions and goals from the face and eyes [71]. MOG is recruited together with mPFC when judging the emotional valence of a dynamic facial expression [72]. Finally, the LPP wave mainly originated from the pMTG in the congruent condition, and from the pSTG in the incongruent condition. The generators of the LPP mismatch effect were manifested in greater activation in the MedFG (Medial Frontal Gyrus, BA 10), a sub area of BA 10, the rostral part of the prefrontal cortex [73], also called the frontal pole. MedFG belongs to the medial prefrontal cortex (mPFC), a region involved in high-level social cognition for deciphering the mental state of others, [74], forming impressions of other people [75] from their faces, as well as the implicit generation and maintenance of an emotional state [76].

Of particular interest, all the brain regions identified by the LORETA in our study were also found by [77] to be recruited when participants had to update their memory of individuals who repeatedly expressed negative facial emotions, and then suddenly changed. Although our results did not evidence activity in the sensorimotor cortex (nor any mismatch effect in the centro-parietal region), it would be interesting to replicate our study using MEPs, along the lines of [33], to examine if sensorimotor mirror-matching contributes to emotion anticipation.

The different sources of the brain component are consistent with the path analysis we conducted in order to investigate the spatio-temporal unfolding of the mismatch effect. In our modeling approach, we assumed that visual object recognition is hierarchically organized in a sequence of cortical activation involving both (feedforward) input from early to higher-level areas, and top-down facilitation (feedback) from frontal regions to temporal regions [78, 79] and even occipital regions [80]. This hypothesis has already been validated using dipole modeling of EEG/MEG data [79], or dynamic causal modeling of fMRI data [81]. Along those lines, Garrido et al., [78] showed that backward connections mediate late components of event-related mismatch responses in an oddball paradigm. Similarly, in social context, there is evidence that visually inferring intentions from a confederate strengthen backward connections from mPFC more strongly than the forward connections from the superior occipital gyrus and MTG to mPFC [81]. New insights into complex social processes, including the perception of facial expressions of emotion, come from studies of white matter tracts (axonal fiber pathways) [82].

Our results regarding the neuroelectric unfolding of the mismatch effect are consistent with the hypothesis that emotional anticipation may reflect top-down emotional processes [25] and constitute a low-level form of the mind-reading mechanism [6]. Path analysis and source localization suggested that MedFG was instrumental in mediating the mismatch effect through top-down influence on both the occipital and temporal regions. This is consistent with the involvement of MedFG in the appraisal of emotions [83, 84]. On the one hand, our path analysis confirmed that the frontal region exerts feedback on the N170 in the temporal region in order to facilitate visual recognition [79]. On the other hand, the mismatch effect on the LPP in the left occipital region is consistent with several results cited in literature. [85] found that when participants are asked to observe a dynamic expression of pain and then indicate the intensity of the expressed pain with their own face rather than simply imitate the observed movement, there is increased activity in the MedFG, left MTG, and left superior occipital gyrus. Moreover, our results are consistent with investigations of structural anatomic connections between brain areas (using diffusion tensor imaging) showing that better social cognition (including Theory of Mind and emotion recognition) is associated with greater axonal coherence (higher axial diffusivity) in the left Inferior Frontal Fascicle (IFOF) and the left uncinate fasciculus (which connects frontal and temporal regions) [86]. Along the same lines, it is now well-established that lesions to the IFOF impair emotion recognition from facial expressions [87].

Regarding low-level forms of mind-reading, from our results we can speculate that congruent test images were checked in more depth to ensure they were truly congruent with the anticipated emotional intensity. By way of contrast, incongruent faces would appear to be more obvious. Furthermore, facial expressions of pain seem to be the most important source of information available to observers to evaluate the authenticity of the pain felt [88]. When an expression is incongruent with an individual's anticipated expression or does not correspond with their expected expression, it could be considered as faked. Therefore, we can assume that automatic emotional anticipation may be useful for prosocial behavior and the detection of trustworthiness, especially for the intermittent viewing of a facial expression. Finally, perceived trustworthiness seems to involve greater empathy [89]; thus, it would be interesting to explore,

in future studies, the link between the individual empathy level and the emotional anticipation process. Moreover, the cognitive anticipation process may be different if an individual perceives a facial expression of pain in an emergency context, and in accordance with his or her experience of this context (e.g., as a clinician, emergency doctor, or firefighter). Whilst clinicians have been shown to underestimate patients' pain [90–92], they may also present a strong degree of emotional anticipation. Such anticipation can be considered adaptive in view of their job constraints. Thus, it remains for future research to explore the neuroelectric correlates of context effect and individual characteristics on the emotional anticipation of pain in others.

Supporting information

S1 File. Experiment 1. (Figure A) Example of behavioral results for one participant in response to the VideoEI90% in the preliminary experiment. This participant presented a significant memory displacement (PSE = 64.8%, further from 90%). In abscissa, the gray brackets illustrate the three TestEIs triplets shown to this participant in the main experiment. In this example, the "Expected Incongruent TestEIs" triplets were equal to 25%, 30%, 35% and 95%, 100%, 105%. The "expected Congruent TestEIs" triplet was equal to 60%, 65% and 70%.
(DOCX)

S2 File. Path analysis model testing. (Figure A) Initial path analysis mediation model testing for the spatio-temporal unfolding of the mismatch effect from Occipital P100 up to Left Occipital LPP. A: this initial model tested the mismatch effect at three components as predictors of the mismatch effect at Left_O_LPP using a serial multiple mediator model with two mediators (F_N170 and T_N170). B: the second model introduced the mismatch effect at F_LPP while considering two simple mediation models arranged in series, with T_N170 and Left_O_LPP as dependent variables. Standardized (β) coefficients (in bold) are provided together with unstandardized coefficients (between parentheses). The unstandardized coefficients are in μV unit, together with significance level of effects as §$p < .10$, $^{*}p < .05$, $^{**}\, p < .01$, $^{***}\, p < .001$. Significant indirect effects are illustrated with thicker lines.
(DOCX)

S3 File. Contains the raw data that were used in order to perform the statistical analyses.
(XLSX)

Author Contributions

Conceptualization: Elise Prigent, Michel-Ange Amorim.

Data curation: Dorian Dozolme, Elise Prigent, Yu-Fang Yang, Michel-Ange Amorim.

Formal analysis: Dorian Dozolme, Elise Prigent, Yu-Fang Yang, Michel-Ange Amorim.

Funding acquisition: Michel-Ange Amorim.

Investigation: Dorian Dozolme, Elise Prigent, Yu-Fang Yang, Michel-Ange Amorim.

Methodology: Dorian Dozolme, Elise Prigent, Michel-Ange Amorim.

Project administration: Dorian Dozolme, Elise Prigent, Michel-Ange Amorim.

Resources: Dorian Dozolme, Elise Prigent, Michel-Ange Amorim.

Software: Dorian Dozolme, Elise Prigent, Michel-Ange Amorim.

Supervision: Dorian Dozolme, Elise Prigent, Michel-Ange Amorim.

Validation: Dorian Dozolme, Elise Prigent, Yu-Fang Yang, Michel-Ange Amorim.

Visualization: Dorian Dozolme, Elise Prigent, Yu-Fang Yang, Michel-Ange Amorim.

Writing – original draft: Dorian Dozolme, Elise Prigent.

Writing – review & editing: Dorian Dozolme, Elise Prigent, Yu-Fang Yang, Michel-Ange Amorim.

References

1. Freyd JJ, Finke RA. Representational momentum. Journal of Experimental Psychology: Learning, Memory, and Cognition. 1984; 10: 126.1.
2. Blättler C, Ferrari V, Didierjean A, van Elslande P, Marmèche E. Can expertise modulate representational momentum? Visual Cognition. 2010; 18: 1253–1273. https://doi.org/10.1080/13506281003737119
3. Hubbard TL. Representational momentum and related displacements in spatial memory: A review of the findings. Psychonomic Bulletin & Review. 2005; 12: 822–851.
4. Yoshikawa S, Sato W. Dynamic facial expressions of emotion induce representational momentum. Cognitive, Affective, & Behavioral Neuroscience. 2008; 8: 25–31.
5. Jarraya M, Amorim M-A, Bardy BG. Optical flow and viewpoint change modulate the perception and memorization of complex motion. Perception & psychophysics. 2005; 67: 951–961.
6. Palumbo L, Jellema T. Beyond face value: does involuntary emotional anticipation shape the perception of dynamic facial expressions? PloS one. 2013; 8: e56003. https://doi.org/10.1371/journal.pone.0056003 PMID: 23409112
7. Freyd JJ. Dynamic mental representations. Psychological review. 1987; 94: 427. PMID: 3317470
8. Craig KD, Versloot J, Goubert L, Vervoort T, Crombez G. Perceiving pain in others: automatic and controlled mechanisms. The Journal of Pain. 2010; 11: 101–108. https://doi.org/10.1016/j.jpain.2009.08.008 PMID: 19962352
9. Ekman P. Facial expressions. Handbook of cognition and emotion. 1999; 16: 301–20.
10. Craig AD. A new view of pain as a homeostatic emotion. Trends in neurosciences. 2003; 26: 303–307. PMID: 12798599
11. Prkachin KM, Hughes E, Schultz I, Joy P, Hunt D. Real-time assessment of pain behavior during clinical assessment of low back pain patients. Pain. 2002; 95: 23–30. PMID: 11790464
12. Prkachin KM, Mercer SR. Pain expression in patients with shoulder pathology: validity, properties and relationship to sickness impact. Pain. 1989; 39: 257–265. PMID: 2616178
13. Williams AC de C. Facial expression of pain, empathy, evolution, and social learning. Behavioral and brain sciences. 2002; 25: 475–480.
14. Damasio AR. Descartes' error. London: Papermac. Macmillan; 1994.
15. Avenanti A, Miniopaluello I, Bufalari I, Aglioti S. The pain of a model in the personality of an onlooker: Influence of state-reactivity and personality traits on embodied empathy for pain. NeuroImage. 2009; 44: 275–283. https://doi.org/10.1016/j.neuroimage.2008.08.001 PMID: 18761092
16. Prkachin KM, Currie NA, Craig KD. Judging nonverbal expressions of pain. Canadian Journal of Behavioural Science/Revue canadienne des sciences du comportement. 1983; 15: 409–421.
17. Sullivan MJ, Thibault P, Savard A, Catchlove R, Kozey J, Stanish WD. The influence of communication goals and physical demands on different dimensions of pain behavior. Pain. 2006; 125: 270–277. https://doi.org/10.1016/j.pain.2006.06.019 PMID: 16860479
18. Poole GD, Craig KD. Judgments of genuine, suppressed, and faked facial expressions of pain. Journal of Personality and Social Psychology. 1992; 63: 797. PMID: 1447693
19. Thornton IM, Kourtzi Z. A matching advantage for dynamic human faces. Perception. 2002; 31: 113–132. https://doi.org/10.1068/p3300 PMID: 11922118
20. Lander K, Bruce V. The role of motion in learning new faces. Visual Cognition. 2003; 10: 897–912.
21. Ambadar Z, Schooler JW, Cohn JF. Deciphering the enigmatic face: the importance of facial dynamics in interpreting subtle facial expressions. Psychological Science. 2005; 16: 403–410. https://doi.org/10.1111/j.0956-7976.2005.01548.x PMID: 15869701
22. Prigent E, Amorim M-A, de Oliveira AM. Representational momentum in dynamic facial expressions is modulated by the level of expressed pain: Amplitude and direction effects. Attention, Perception, & Psychophysics. 2018; 80: 82–93. https://doi.org/10.3758/s13414-017-1422-6 PMID: 28956325

23. Courgeon M, Amorim MA, Giroux C, Martin JC. Do users anticipate emotion dynamics in facial expressions of a virtual character. Proceedings of the 23rd international conference on computer animation and social agents (CASA 2010), Saint Malo, France. 2010.
24. Thornton IM. Representational momentum and the human face: an empirical note. Xjenza 2014; 2(2), 9, 101–110. https://doi.org/10.7423/XJENZA.2014.2.09
25. Jellema T, Pecchinenda A, Palumbo L, Tan EG. Biases in the perception and affective valence of neutral facial expressions induced by the immediate perceptual history. Visual Cognition. 2011; 19: 616–634.
26. Niedenthal PM, Barsalou LW, Ric F, Krauth-Gruber S. Embodiment in the acquisition and use of emotion knowledge. Emotion and consciousness. 2005; 21–50.
27. Wicker B, Keysers C, Plailly J, Royet J-P, Gallese V, Rizzolatti G. Both of us disgusted in My insula: the common neural basis of seeing and feeling disgust. Neuron. 2003; 40: 655–664. PMID: 14642287
28. Botvinick M, Jha AP, Bylsma LM, Fabian SA, Solomon PE, Prkachin KM. Viewing facial expressions of pain engages cortical areas involved in the direct experience of pain. Neuroimage. 2005; 25: 312–319. https://doi.org/10.1016/j.neuroimage.2004.11.043 PMID: 15734365
29. Avenanti A, Bueti D, Galati G, Aglioti SM. Transcranial magnetic stimulation highlights the sensorimotor side of empathy for pain. Nature Neuroscience. 2005; 8: 955–960. https://doi.org/10.1038/nn1481 PMID: 15937484
30. Avenanti A, Paluello IM, Bufalari I, Aglioti SM. Stimulus-driven modulation of motor-evoked potentials during observation of others' pain. NeuroImage. 2006; 32: 316–324. https://doi.org/10.1016/j.neuroimage.2006.03.010 PMID: 16675270
31. Bucchioni G, Fossataro C, Cavallo A, Mouras H, Neppi-Modona M, Garbarini F. Empathy or Ownership? Evidence from Corticospinal Excitability Modulation during Pain Observation. Journal of Cognitive Neuroscience. 2016; 28: 1760–1771. https://doi.org/10.1162/jocn_a_01003 PMID: 27378331
32. Borgomaneri S, Vitale F, Gazzola V, Avenanti A. Seeing fearful body language rapidly freezes the observer's motor cortex. Cortex. 2015; 65: 232–245. https://doi.org/10.1016/j.cortex.2015.01.014 PMID: 25835523
33. Bufalari I, Aprile T, Avenanti A, Di Russo F, Aglioti SM. Empathy for Pain and Touch in the Human Somatosensory Cortex. Cerebral Cortex. 2007; 17: 2553–2561. https://doi.org/10.1093/cercor/bhl161 PMID: 17205974
34. Haxby JV, Gobbini MI. Distributed neural systems for face perception. The Oxford Handbook of Face Perception; 2011.
35. Reicherts P, Wieser MJ, Gerdes AB, Likowski KU, Weyers P, Mühlberger A, et al. Electrocortical evidence for preferential processing of dynamic pain expressions compared to other emotional expressions. PAIN®. 2012; 153: 1959–1964.
36. Furl N, van Rijsbergen NJ, Kiebel SJ, Friston KJ, Treves A, Dolan RJ. Modulation of perception and brain activity by predictable trajectories of facial expressions. Cerebral Cortex. 2010; 20: 694–703. https://doi.org/10.1093/cercor/bhp140 PMID: 19617291
37. Pineda JA. Sensorimotor cortex as a critical component of an "extended" mirror neuron system: Does it solve the development, correspondence, and control problems in mirroring? Behavioral and Brain Functions. 2008; 4: 47. https://doi.org/10.1186/1744-9081-4-47 PMID: 18928566
38. Ekman P, Friesen WV,.Facial action coding system : A technique for the measurement of facial movement. Consulting Psychologists Press, Palo Alto. 1978.
39. Prkachin KM. The consistency of facial expressions of pain: a comparison across modalities. Pain. 1992; 51: 297–306. PMID: 1491857
40. Oliveira AM, De Sá Teixeira NA, Oliveira MP, Breda SJ, Da Fonseca I. Algebraic integration models of facial features of expression: A case made for pain. Teorie & Modelli Rivista di Storia e Metodologia della Psicologia. 2007; 12: 155–166.
41. Macar F, Vitton N. CNV and reaction time task in man: effects of inter-stimulus interval contingencies. Neuropsychologia. 1980; 18: 585–590. PMID: 7443023
42. Lin H, Gao H, You J, Liang J, Ma J, Yang N, et al. Larger N2 and smaller early contingent negative variation during the processing of uncertainty about future emotional events. International Journal of Psychophysiology. 2014; 94: 292–297. https://doi.org/10.1016/j.ijpsycho.2014.10.004 PMID: 25312204
43. Stone JV. Using reaction times and binary responses to estimate psychophysical performance: an information theoretic analysis. Frontiers in Neuroscience. 2014; 8:35. https://doi.org/10.3389/fnins.2014.00035 PMID: 24624053
44. Ibanez A, Melloni M, Huepe D, Helgiu E, Rivera-Rei A, Canales-Johnson A, et al. What event-related potentials (ERPs) bring to social neuroscience? Social Neuroscience. 2012; 7: 632–649. https://doi.org/10.1080/17470919.2012.691078 PMID: 22642412

45. Kutas M, Federmeier KD. Thirty Years and Counting: Finding Meaning in the N400 Component of the Event-Related Brain Potential (ERP). Annual Review of Psychology. 2011; 62: 621–647. https://doi.org/10.1146/annurev.psych.093008.131123 PMID: 20809790

46. Handy TC. Event-related potentials: A methods handbook. MIT press; 2005.

47. Pascual-Marqui RD, Michel CM, Lehmann D. Low resolution electromagnetic tomography: a new method for localizing electrical activity in the brain. International Journal of psychophysiology. 1994; 18: 49–65. PMID: 7876038

48. Keselman HJ. Testing treatment effects in repeated measures designs: an update for psychophysiological researchers. Psychophysiology. 1998; 35: 470–478. PMID: 9643062

49. Wilks SS. Certain generalizations in the analysis of variance. Biometrika. 1932; 471–494.

50. Dien J, Santuzzi AM. 4 Application of Repeated Measures ANOVA to High-Density ERP. Event-related potentials: A methods handbook. 2004; 57–81.

51. Hayes AF. Introduction to mediation, moderation, and conditional process analysis: A regression-based approach. Guilford Publications; 2013.

52. Rousselet GA, Foxe JJ, Bolam JP. A few simple steps to improve the description of group results in neuroscience. European Journal of Neuroscience. 2016; 44: 2647–2651. https://doi.org/10.1111/ejn.13400 PMID: 27628462

53. Dozolme D, Brunet-Gouet E, Passerieux C, Amorim M-A. Neuroelectric Correlates of Pragmatic Emotional Incongruence Processing: Empathy Matters. PloS one. 2015; 10: e0129770. https://doi.org/10.1371/journal.pone.0129770 PMID: 26067672

54. Prieto del Val L, Cantero JL, Baena D, Atienza M. Damage of the temporal lobe and APOE status determine neural compensation in mild cognitive impairment. Cortex. 2018; 101: 136–153. https://doi.org/10.1016/j.cortex.2018.01.018 PMID: 29475078

55. Lim S-L, Padmala S, Pessoa L. Segregating the significant from the mundane on a moment-to-moment basis via direct and indirect amygdala contributions. Proceedings of the National Academy of Sciences. 2009; 106: 16841–16846. https://doi.org/10.1073/pnas.0904551106 PMID: 19805383

56. Miceli M, Castelfranchi C. Expectancy and Emotion. Oxford University Press, USA; 2015.

57. Craig KD, Patrick CJ. Facial expression during induced pain. Journal of personality and social psychology. 1985; 48: 1080. PMID: 3989673

58. Craig KD, Hyde SA, Patrick CJ. Genuine, suppressed and faked facial behavior during exacerbation of chronic low back pain. Pain. 1991; 46: 161–171. PMID: 1836259

59. Niedenthal PM, Mermillod M, Maringer M, Hess U. The Simulation of Smiles (SIMS) model: Embodied simulation and the meaning of facial expression. Behavioral and brain sciences. 2010; 33: 417–433. https://doi.org/10.1017/S0140525X10000865 PMID: 21211115

60. Hubbard TL. Forms of momentum across space: Representational, operational, and attentional. Psychonomic Bulletin & Review. 2014; 21: 1371–1403.

61. Radvansky GA, Zacks JM. Event cognition. Oxford University Press; 2014.

62. Courtney JR, Hubbard TL. Spatial memory and explicit knowledge: An effect of instruction on representational momentum. The Quarterly Journal of Experimental Psychology. 2008; 61: 1778–1784. https://doi.org/10.1080/17470210802194217 PMID: 18609396

63. Ruppel SE, Fleming CN, Hubbard TL. Representational momentum is not (totally) impervious to error feedback. Canadian Journal of Experimental Psychology/Revue canadienne de psychologie expérimentale. 2009; 63: 49–58. https://doi.org/10.1037/a0013980 PMID: 19271816

64. Hietanen JK, Astikainen P. N170 response to facial expressions is modulated by the affective congruency between the emotional expression and preceding affective picture. Biological Psychology. 2013; 92: 114–124. https://doi.org/10.1016/j.biopsycho.2012.10.005 PMID: 23131616

65. Schweinberger SR, Pickering EC, Jentzsch I, Burton AM, Kaufmann JM. Event-related brain potential evidence for a response of inferior temporal cortex to familiar face repetitions. Cognitive Brain Research. 2002; 14: 398–409. PMID: 12421663

66. Schweinberger SR, Neumann MF. Repetition effects in human ERPs to faces. Cortex. 2016; 80: 141–153. https://doi.org/10.1016/j.cortex.2015.11.001 PMID: 26672902

67. Wirth BE, Fisher K, Towler J, Eimer M. Facial misidentifications arise from the erroneous activation of visual face memory. Neuropsychologia. 2015; 77: 387–399. https://doi.org/10.1016/j.neuropsychologia.2015.09.021 PMID: 26384776

68. Fisher K, Towler J, Eimer M. Facial identity and facial expression are initially integrated at visual perceptual stages of face processing. Neuropsychologia. 2016; 80: 115–125. https://doi.org/10.1016/j.neuropsychologia.2015.11.011 PMID: 26581627

69. Chronaki G, Broyd SJ, Garner M, Benikos N, Thompson MJJ, Sonuga-Barke EJS, et al. The Moderating Effect of Self-Reported State and Trait Anxiety on the Late Positive Potential to Emotional Faces in 6–11-Year-Old Children. Frontiers in Psychology. 2018; 9:125. https://doi.org/10.3389/fpsyg.2018.00125 PMID: 29515476

70. Thirioux B, Mercier MR, Blanke O, Berthoz A. The cognitive and neural time course of empathy and sympathy: An electrical neuroimaging study on self–other interaction. Neuroscience. 2014; 267: 286–306. https://doi.org/10.1016/j.neuroscience.2014.02.024 PMID: 24583040

71. Yordanova YN, Duffau H, Herbet G. Neural pathways subserving face-based mentalizing. Brain Structure and Function. 2017; 222: 3087–3105. https://doi.org/10.1007/s00429-017-1388-0 PMID: 28243761

72. Skerry AE, Saxe R. A Common Neural Code for Perceived and Inferred Emotion. Journal of Neuroscience. 2014; 34: 15997–16008. https://doi.org/10.1523/JNEUROSCI.1676-14.2014 PMID: 25429141

73. Gilbert SJ, Gonen-Yaacovi G, Benoit RG, Volle E, Burgess PW. Distinct functional connectivity associated with lateral versus medial rostral prefrontal cortex: A meta-analysis. NeuroImage. 2010; 53: 1359–1367. https://doi.org/10.1016/j.neuroimage.2010.07.032 PMID: 20654722

74. Frith CD, Frith U. Social Cognition in Humans. Current Biology. 2007; 17: R724–R732. https://doi.org/10.1016/j.cub.2007.05.068 PMID: 17714666

75. Mitchell JP, Neil Macrae C, Banaji MR. Forming impressions of people versus inanimate objects: Social-cognitive processing in the medial prefrontal cortex. NeuroImage. 2005; 26: 251–257. https://doi.org/10.1016/j.neuroimage.2005.01.031 PMID: 15862225

76. Waugh CE, Lemus MG, Gotlib IH. The role of the medial frontal cortex in the maintenance of emotional states. Social Cognitive and Affective Neuroscience. 2014; 9: 2001–2009. https://doi.org/10.1093/scan/nsu011 PMID: 24493835

77. Nashiro K, Sakaki M, Nga L, Mather M. Age-related similarities and differences in brain activity underlying reversal learning. Frontiers in Integrative Neuroscience. 2013; 7:37. https://doi.org/10.3389/fnint.2013.00037 PMID: 23750128

78. Garrido MI, Kilner JM, Kiebel SJ, Friston KJ. Evoked brain responses are generated by feedback loops. Proceedings of the National Academy of Sciences. 2007; 104: 20961–20966. https://doi.org/10.1073/pnas.0706274105 PMID: 18087046

79. Ahlfors SP, Jones SR, Ahveninen J, Hämäläinen MS, Belliveau JW, Bar M. Direction of magnetoencephalography sources associated with feedback and feedforward contributions in a visual object recognition task. Neuroscience Letters. 2015; 585: 149–154. https://doi.org/10.1016/j.neulet.2014.11.029 PMID: 25445356

80. Pantazatos SP, Yanagihara TK, Zhang X, Meitzler T, Hirsch J. Frontal–Occipital Connectivity During Visual Search. Brain Connectivity. 2012; 2: 164–175. https://doi.org/10.1089/brain.2012.0072 PMID: 22708993

81. Hillebrandt H, Dumontheil I, Blakemore S-J, Roiser JP. Dynamic causal modelling of effective connectivity during perspective taking in a communicative task. Neuroimage. 2013; 76: 116–124. https://doi.org/10.1016/j.neuroimage.2013.02.072 PMID: 23507383

82. Wang Y, Olson IR. The Original Social Network: White Matter and Social Cognition. Trends in Cognitive Sciences. 2018; https://doi.org/10.1016/j.tics.2018.03.005 PMID: 29628441

83. Etkin A, Egner T, Kalisch R. Emotional processing in anterior cingulate and medial prefrontal cortex. Trends in Cognitive Sciences. 2011; 15: 85–93. https://doi.org/10.1016/j.tics.2010.11.004 PMID: 21167765

84. Tseng W-L, Thomas LA, Harkins E, Stoddard J, Zarate CA, Pine DS, et al. Functional connectivity during masked and unmasked face emotion processing in bipolar disorder. Psychiatry Research: Neuroimaging. 2016; 258: 1–9. https://doi.org/10.1016/j.pscychresns.2016.10.006 PMID: 27814457

85. Budell L, Kunz M, Jackson PL, Rainville P. Mirroring Pain in the Brain: Emotional Expression versus Motor Imitation. Lamm C, editor. PLOS ONE. 2015; 10: e0107526. https://doi.org/10.1371/journal.pone.0107526 PMID: 25671563

86. Jalbrzikowski M, Villalon-Reina JE, Karlsgodt KH, Senturk D, Chow C, Thompson PM, et al. Altered white matter microstructure is associated with social cognition and psychotic symptoms in 22q11.2 microdeletion syndrome. Frontiers in Behavioral Neuroscience. 2014; 8. https://doi.org/10.3389/fnbeh.2014.00393 PMID: 25426042

87. Philippi CL, Mehta S, Grabowski T, Adolphs R, Rudrauf D. Damage to Association Fiber Tracts Impairs Recognition of the Facial Expression of Emotion. Journal of Neuroscience. 2009; 29: 15089–15099. https://doi.org/10.1523/JNEUROSCI.0796-09.2009 PMID: 19955360

88. Martel MO, Thibault P, Sullivan MJ. Judgments about pain intensity and pain genuineness: the role of pain behavior and judgmental heuristics. The Journal of Pain. 2011; 12: 468–475. https://doi.org/10.1016/j.jpain.2010.10.010 PMID: 21296030

89. Sessa P, Meconi F. Perceived trustworthiness shapes neural empathic responses toward others' pain. Neuropsychologia. 2015; 79: 97–105. https://doi.org/10.1016/j.neuropsychologia.2015.10.028 PMID: 26514617

90. Marquié L, Raufaste E, Lauque D, Mariné C, Ecoiffier M, Sorum P. Pain rating by patients and physicians: evidence of systematic pain miscalibration. Pain. 2003; 102: 289–296. PMID: 12670671

91. Kappesser J, de C Williams AC. Pain and negative emotions in the face: judgements by health care professionals. Pain. 2002; 99: 197–206. PMID: 12237197

92. Prigent E, Amorim M-A, Leconte P, Pradon D. Perceptual weighting of pain behaviours of others, not information integration, varies with expertise. European Journal of Pain. 2014; 18: 110–119. https://doi.org/10.1002/j.1532-2149.2013.00354.x PMID: 23821536
